# Dual Targeting of BRAF and mTOR Signaling in Melanoma Cells with Pyridinyl Imidazole Compounds

**DOI:** 10.3390/cancers12061516

**Published:** 2020-06-10

**Authors:** Veronika Palušová, Tereza Renzová, Amandine Verlande, Tereza Vaclová, Michaela Medková, Linda Cetlová, Miroslava Sedláčková, Hana Hříbková, Iva Slaninová, Miriama Krutá, Vladimír Rotrekl, Hana Uhlířová, Aneta Křížová, Radim Chmelík, Pavel Veselý, Michaela Krafčíková, Lukáš Trantírek, Kay Oliver Schink, Stjepan Uldrijan

**Affiliations:** 1 Faculty of Medicine, Masaryk University, Kamenice 753/5, 625 00 Brno, Czech Republic; ver.palusova@mail.muni.cz (V.P.); spoustova@med.muni.cz (T.R.); verlande.amandine@gmail.com (A.V.); vaclovat@gmail.com (T.V.); 436779@mail.muni.cz (M.M.); lindacetlova@seznam.cz (L.C.); msedl@med.muni.cz (M.S.); hribkova@med.muni.cz (H.H.); ipokorna@med.muni.cz (I.S.); kruta.miriama@gmail.com (M.K.); vrotrekl@med.muni.cz (V.R.); 2 International Clinical Research Center, St. Anne’s University Hospital Brno, Pekařská 664/53, 656 91 Brno, Czech Republic; 3 Institute of Physical Engineering, Faculty of Mechanical Engineering, Brno University of Technology, Technická 2896/2, 616 69 Brno, Czech Republic; hana.uhlirova@ceitec.vutbr.cz (H.U.); radim.chmelik@ceitec.vutbr.cz (R.C.); 4 CEITEC—Central European Institute of Technology, Brno University of Technology, Purkyňova 656/123, 612 00 Brno, Czech Republic; aneta.krizova@ceitec.vutbr.cz (A.K.); pavel.vesely@ceitec.vutbr.cz (P.V.); 5 National Centre for Biomolecular Research, Masaryk University, Kamenice 753/5, 625 00 Brno, Czech Republic; 393655@mail.muni.cz; 6 CEITEC—Central European Institute of Technology, Masaryk University, Kamenice 753/5, 625 00 Brno, Czech Republic; trantirek@ceitec.muni.cz; 7 Centre for Cancer Cell Reprogramming, Faculty of Medicine, University of Oslo, Montebello, N-0379 Oslo, Norway; Kay.Oliver.Schink@rr-research.no; 8 Department of Molecular Cell Biology, Institute for Cancer Research, Oslo University Hospital, Montebello, N-0379 Oslo, Norway

**Keywords:** melanoma, BRAF V600E, BRAF inhibitor, small molecule drug, pyridinyl imidazole, endosome, lysosome, mTORC1, ER stress

## Abstract

BRAF inhibitors can delay the progression of metastatic melanoma, but resistance usually emerges, leading to relapse. Drugs simultaneously targeting two or more pathways essential for cancer growth could slow or prevent the development of resistant clones. Here, we identified pyridinyl imidazole compounds SB202190, SB203580, and SB590885 as dual inhibitors of critical proliferative pathways in human melanoma cells bearing the V600E activating mutation of BRAF kinase. We found that the drugs simultaneously disrupt the BRAF V600E-driven extracellular signal-regulated kinase (ERK) mitogen-activated protein kinase (MAPK) activity and the mechanistic target of rapamycin complex 1 (mTORC1) signaling in melanoma cells. Pyridinyl imidazole compounds directly inhibit BRAF V600E kinase. Moreover, they interfere with the endolysosomal compartment, promoting the accumulation of large acidic vacuole-like vesicles and dynamic changes in mTOR signaling. A transient increase in mTORC1 activity is followed by the enrichment of the Ragulator complex protein p18/LAMTOR1 at contact sites of large vesicles and delocalization of mTOR from the lysosomes. The induced disruption of the endolysosomal pathway not only disrupts mTORC1 signaling, but also renders melanoma cells sensitive to endoplasmic reticulum (ER) stress. Our findings identify new activities of pharmacologically relevant small molecule compounds and provide a biological rationale for the development of anti-melanoma therapeutics based on the pyridinyl imidazole core.

## 1. Introduction

Malignant melanoma is aggressive cancer affecting the skin and other tissues where pigment-producing melanocytes reside. Although melanoma accounts for less than 5% of all dermatologic tumors, it is responsible for approximately 80% of all deaths from skin cancers [1]. The main risk factor for the development of melanoma is overexposure to solar UV radiation. The UV light can induce cancer-promoting mutations that can stimulate melanocyte growth independently from external stimuli [2,3]. Most human melanomas can be placed into two subgroups based on the type of mutation driving the ERK MAPK signaling pathway and cell proliferation [4,5]. About a half of melanoma patients carry a potent activating mutation V600E in the BRAF kinase, which renders the normally dimeric BRAF kinase enzymatically active as a monomer, independent of upstream signaling. The second-largest group of melanoma patients bears activating mutations in the NRAS protein.

The crucial role of BRAF-driven oncogenic ERK signaling in melanoma stimulated the preclinical and clinical development of a large number of structurally different RAF inhibitors [6]. Targeting mutant BRAF can lead to a strong response in melanoma patients. Still, unfortunately, the cancers commonly acquire other pro-survival mutations compensating for the depletion of BRAF activity [7] and causing resistance to treatment [8]. In BRAF inhibitor-resistant melanoma cells, a combination of MEK inhibitors with phosphatidylinositol 3-kinase (PI3K) or mechanistic target of rapamycin (mTOR) inhibitors can significantly decrease cancer cell survival [9,10]. Furthermore, in mouse models of BRAF-mutated melanoma, inhibition of PI3K cooperated with ERK pathway inhibition to forestall the onset of MAPK pathway inhibitor resistance [11].

Activation of receptor tyrosine kinases by growth factors stimulates both MAPK and PI3K/AKT/mTOR signaling pathways to coordinate cell growth, proliferation, and survival [12]. The mTOR serine/threonine protein kinase is a core component of two functionally distinct protein complexes—mTOR complex 1 (mTORC1) and 2 (mTORC2). Complex 1 balances the anabolic and catabolic processes in response to growth factors with ATP, oxygen, and nutrient availability. When active, mTORC1 stimulates proteosynthesis and inhibits catabolic processes such as autophagy [13]. Small GTPases Rheb (Ras homolog enriched in brain) and Rag mediate the activation of mTORC1 [14]. To activate the mTORC1 complex, Rheb must be in a GTP-bound state and proximity to mTORC1 [15]. Insufficient growth factor signaling, low glucose conditions, or low oxygen conditions promote the dissociation of the TSC1/2 protein complex. Released TSC2 causes a switch of Rheb into its inactive GDP-bound state, preventing the activation of mTORC1 [16,17]. Interestingly, TSC2 translocation to the lysosome appears to be a universal cellular response to stress stimuli [18].

Critical factors enabling the mTOR complex 1 anchoring to endomembranes are the Rag family of small GTPases and a scaffold protein complex called Ragulator [19,20]. Four related Rag GTPases, RagA, RagB, RagC, and RagD, are expressed in mammalian cells. They form RagA/B–RagC/D heterodimers, in which RagA or RagB physically interact with RagC or RagD [20,21]. The recruitment of mTORC1 to the lysosome depends on the nucleotide-bound state of Rags, which form a docking site for Raptor, an essential subunit of mTORC1 [22]. In amino acid-starved conditions, RagA/B are bound to GDP, which is rapidly exchanged with GTP when amino acid levels are restored [20,23]. The sensing of amino acid levels takes place in the lysosomal lumen, and individual amino acids have their specific sensors, which act upstream of Rag GTPases [24,25].

The PI3K/AKT/mTOR and RAF/MEK/ERK signaling cascades form numerous feedback loops and interconnect at multiple points of crosstalk. Inhibition of one pathway can be partially compensated by the enhanced activity of the other, implying that dual targeting of both pathways may improve treatment efficacy and lead to better clinical outcomes [26]. In the current study, we show that pyridinyl imidazole compounds are capable of simultaneously targeting the BRAF oncogene and mTORC1 signaling in human melanoma cells. They directly inhibit the BRAF kinase activity and, at the same time, interfere with the endolysosomal compartment, leading to a loss of mTORC1 lysosomal localization and activity. The pyridinyl imidazole compounds suppress the growth and proliferation of melanoma cells and sensitize them to additional stress stimuli. These findings provide a biological rationale for further development of pyridinyl imidazole anti-melanoma drugs.

## 2. Results

### 2.1. Pyridinyl Imidazole p38 MAPK Inhibitors Disrupt BRAF V600E-Driven ERK Signaling in Human Melanoma Cells

**Figure 1:**
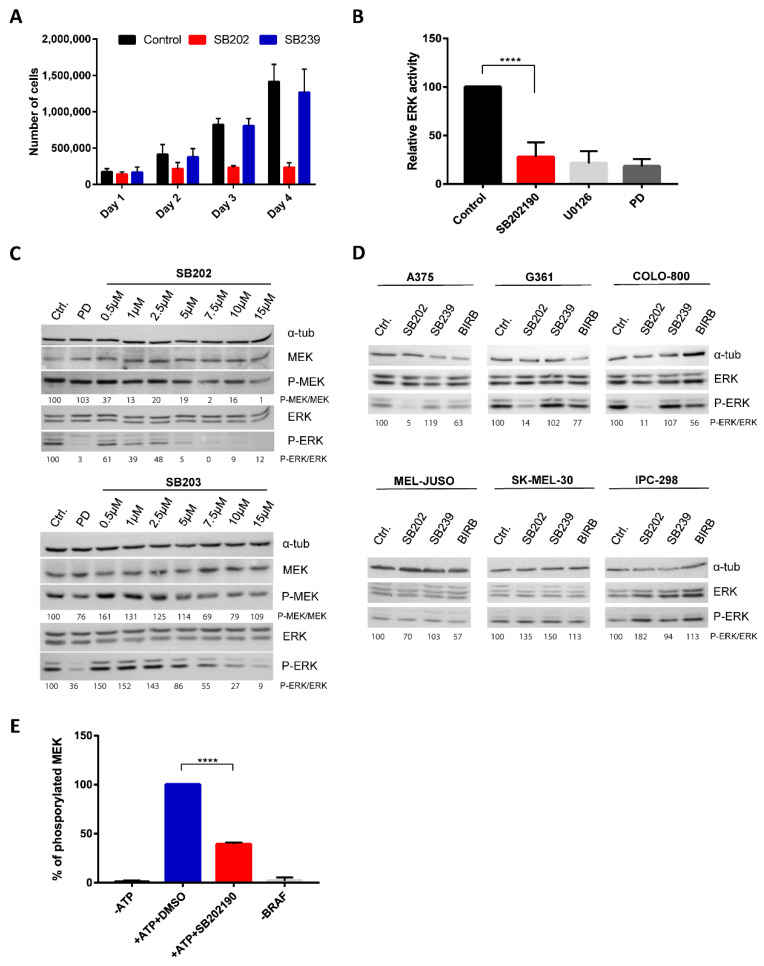
Pyridinyl imidazole p38 mitogen-activated protein kinase (MAPK) inhibitors SB202190 and SB203580 disrupt mutant BRAF kinase activity in human melanoma cells. (**A**) The proliferation of A375 cells was measured by flow cytometry during 4-day cultivation. The number of cells was compared between controls (DMSO-treated) and cells treated with SB202190 (SB202; 15 µM) and SB239063 (SB239; 15 µM). The presented data (mean + SD) were obtained in three independent experiments. (**B**) Relative ERK activity in A375 cells stably transfected with an ERK activity luciferase reporter plasmid. Cells were treated for 24 h with SB202190 (10 µM) and MEK inhibitors U0126 (10 µM) and PD184352 (PD; 1 µM). Three independent experiments were performed. Results are presented as mean relative ERK activity + SD. (**C**) Levels of phosphorylated MEK and ERK kinase were analyzed by Western blot. A375 cells were treated for one hour with increasing concentrations of SB202190 (SB202) and SB203580 (SB203). MEK inhibitor PD184352 (PD; 100 nM) was used as a positive control. The relative ratio between P-ERK and the total ERK levels is also indicated for each sample. (**D**) ERK activity was analyzed by Western blot in melanoma cell lines bearing BRAF (A375, G361, COLO-800) or NRAS (MEL-JUSO, SK-MEL-30, IPC-298) mutations. Cells were treated for 24 h with the p38 inhibitors SB202190 (SB202; 10 µM), SB239063 (SB239; 10 µM), and BIRB796 (BIRB; 10 µM). The relative ratio between P-ERK and the total ERK levels is also indicated for each sample. (**E**) In vitro BRAF kinase assay using kinase-dead MEK as a substrate and endogenous V600E BRAF kinase immunoprecipitated from A375 cells. SB202190 was used at 5 µM. Data were obtained in three independent experiments. Results are presented as relative values, mean + SD. **** denotes *p* < 0.0001.

Pyridinyl imidazole inhibitors SB202190 and SB203580 have been widely used in biomedical research as selective chemical probes for p38 MAPK biological activity, despite reports suggesting additional kinase targets and cell type-specific p38-independent effects of these compounds [27,28,29,30]. We analyzed the biological activity of SB202190 in A375 melanoma cells and found that SB202190, but not a structurally distinct p38 inhibitor SB239063, strongly affected the growth of melanoma cell cultures, suggesting a p38-independent antiproliferative effect of the pyridinyl imidazole compound in human melanoma cells (Figure 1A).

The most common drivers of melanoma proliferation are NRAS and BRAF mutations, constitutively activating the ERK MAPK pathway in about 80% of tumors [4,5]. Interestingly, some reports suggested that pyridinyl imidazole compounds could activate ERK signaling by promoting CRAF (RAF-1) activity [31,32,33]. A small-molecule library screen using bioluminescence resonance energy transfer-based biosensors identified SB202190 and SB203580 as potent activators of RAF dimerization, which might explain the reported ERK pathway activation in response to SB203580 [34]. We, therefore, tested the possibility that the pyridinyl imidazole p38 inhibitors could directly modulate RAF kinase activity and ERK signaling in melanoma cells. We analyzed ERK-dependent transcription in A375 cells, bearing the most common activating mutation of BRAF kinase (V600E), stably transfected with a recently developed ERK activity luciferase reporter construct [35]. Surprisingly, we found that SB202190 strongly inhibited ERK-driven luciferase activity in this system, as potently as MEK kinase inhibitors U0126 and PD184352 that were used as positive controls (Figure 1B). 

Next, we treated A375 cells with increasing concentrations of SB202190 or SB203580 and analyzed ERK pathway activity by Western blotting, using MEK and ERK phospho-specific antibodies. Specific MEK inhibitor PD184352 served as a positive control. Both pyridinyl imidazole compounds induced a dose-dependent decrease in the levels of active ERK and MEK kinases (Figure 1C). The experiment was repeated three times (additional Western blots are available in Figure S1), and we determined the relative P-MEK/MEK and P-ERK/ERK ratios between phosphorylated (active) and total MEK and ERK kinase levels. The results presented in Figure S2 indicate that both compounds could inhibit ERK pathway activity in A375 cells, but SB202190 affected the pathway more potently than SB203580. The fact that both MEK and ERK activity was decreased suggested that the pyridinyl imidazole compounds target the ERK signaling pathway upstream of MEK kinase.

The inhibitory effect of SB202190 on ERK activity was observed in human melanoma cell lines carrying BRAF V600E mutation (A375, G361, Colo-800), but not in melanoma cells with NRAS mutations (MEL-JUSO, SK-MEL-30, IPC-298) (Figure 1D). This result indicated that pyridinyl imidazole p38 inhibitors might act as inhibitors of mutant BRAF, but not wild type CRAF kinase, which activates MEK in cells bearing mutated NRAS. Importantly, two structurally unrelated small-molecule p38 inhibitors SB239063 and BIRB796 did not affect ERK activity in melanoma cells (Figure 1D). The results of two additional independent replicates of this experiment are available in Figure S1.

Next, we performed an in vitro BRAF kinase activity assay using a recombinant kinase-dead MEK protein as a substrate. Three independent experiments were performed, and the levels of MEK phosphorylation were determined by Western blotting and quantified using ImageJ/Fiji (https://imagej.net/Fiji). The results presented in Figure 1E suggest that SB202190 could inhibit the activity of endogenous BRAF V600E protein immunoprecipitated from A375 melanoma cells.

The possibility that the p38 MAPK inhibitors SB202190 and SB203580 might target mutant BRAF kinase was indirectly supported by the fact that a structurally related pyridinyl imidazole derivative SB590885 was developed as a BRAF-specific inhibitor [36]. When we compared in the 3-(4,5-dimethylthiazol-2-yl)-2,5-diphenyltetrazolium bromide (MTT) assays the effect of SB202190 and SB590885 on the proliferation of a panel of melanoma cell lines, as expected, we observed that BRAF-mutated melanoma cell lines were more sensitive to the compounds than NRAS-mutated melanoma cells (Figure S3). BRAF-inhibitor vemurafenib served as a positive control. Interestingly, higher concentrations of SB590885 also negatively affected the growth of NRAS-mutated cell lines, indicating the possibility of additional, BRAF-independent, cytotoxic activity of the pyridinyl imidazole compounds in melanoma cells (Figure S3).

### 2.2. SB202190-Induced Vacuoles in Melanoma Cells Have an Endocytic Origin 

**Figure 2:**
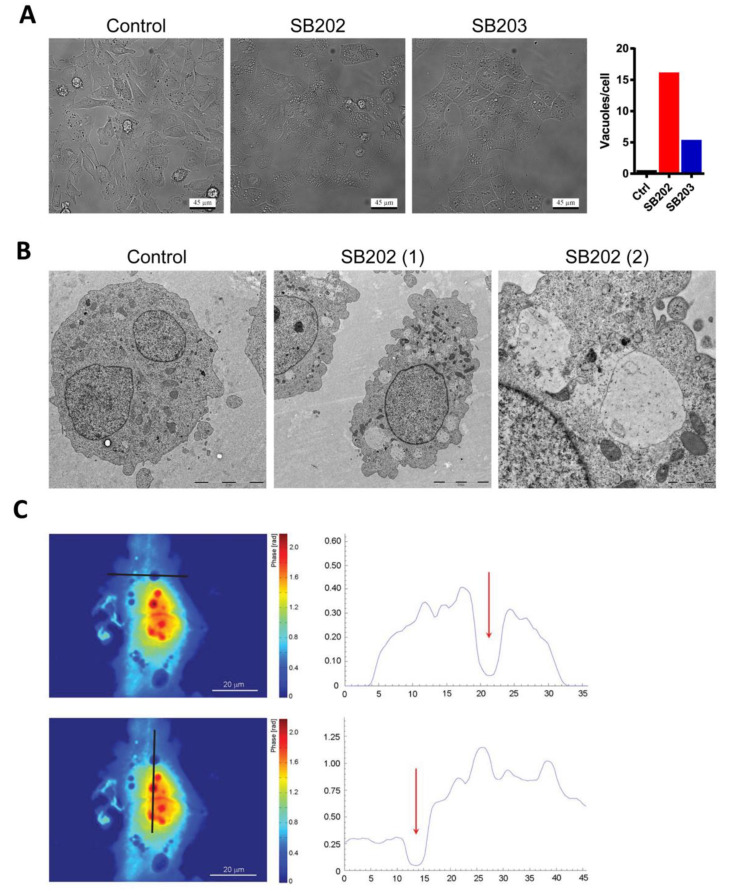
Pyridinyl imidazole compounds induce vacuolization of cytoplasm in BRAF-mutated human melanoma cells. A375 cells were treated with DMSO (control), SB202190 (SB202; 10 μM), and SB203580 (SB203; 10 μM). (**A**) Phase-contrast light microscopy of living cells after 12 h treatment with pyridinyl imidazole p38 MAPK inhibitors. Scale bar: 45 µm. The representative graph shows the mean number of vacuoles per cell quantified using ImageJ/Fiji (find maxima—bright spots above a certain threshold). Dying rounded cells were excluded from the analysis. Similar results were obtained in three independent experiments. (**B**) The content of vacuole-like vesicles was visualized by electron microscopy 24 h post-treatment with SB202190 (SB202). Control and SB202(1)—scale bar 5 µm. SB202(2)—scale bar 1 µm. Two independent experiments showed similar results. (**C**) Quantitative phase-imaging analysis of cellular dry mass in melanoma cells treated for 12 h with SB202190. The zero level of dry mass density was defined as the density of the observation medium. The areal density distribution of the dry mass was quantified in profiles (right panels) indicated by dark lines. Red arrows highlight the position of vacuoles. Two independent digital holography microscopy experiments showed similar results.

Among the effects reported for the p38 MAPK inhibitors, SB202190 and SB203580, was the formation of large vacuole-like structures. Some reports linked the phenotype to the disruption of autophagy, which was later shown to be p38-independent [30,37]. In our experiments, both compounds induced strong cytoplasmic vacuolization in A375 melanoma cells (Figure 2A). We, therefore, analyzed in detail this phenotype and its possible contribution to the growth-inhibitory activity of the pyridinyl imidazole drugs in melanoma cells. Analysis of individual frames of a time-lapse recording from a phase-contrast microscope revealed that the large vacuoles induced by SB202190 in melanoma cells might form by the fusion of smaller vesicles (Figure S4). Electron microscopy analysis of SB202190-treated A375 cells showed that the large vacuoles are mostly devoid of dense structures (Figure 2B). The low mass density of the vacuole-like structures was also confirmed using quantitative phase imaging (Figure 2C). 

**Figure 3:**
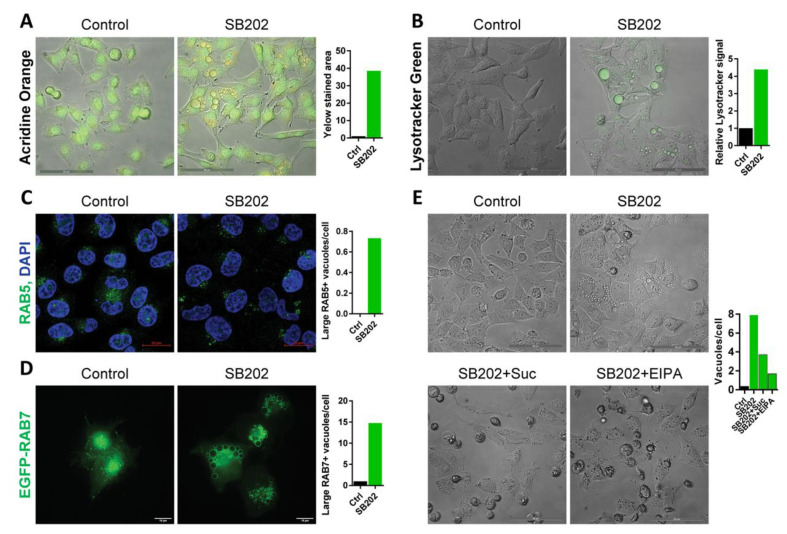
Vacuole-like vesicles induced by pyridinyl imidazole compounds in melanoma cells have an endolysosomal origin. A375 cells were treated with SB202190 (SB202; 15 μM) or the equivalent amount of vehicle (DMSO) in control. (**A**) Cells were treated with SB202190 for 20 h and stained with acridine orange (5 µg/mL) for 15 min. Scale bar: 50 µm. The graph shows the relative change in the yellow stained area in response to SB202190. Similar results were obtained in two independent experiments. (**B**) Cells were treated with SB202190 for 20 h and stained with LysoTracker Green (50 nM) for 15 min. Scale bar: 50 µm. The graph shows the relative change of the LysoTracker Green signal in response to SB202190. Similar results were obtained in two independent experiments. (**C**) The confocal microscopy detection of endogenous early endosomal marker RAB5 after 24 h treatment with SB202190. Scale bar: 20 µm. The graph shows the number of RAB5-positive vacuoles per cell with a diameter larger than 1 µm. Similar results were obtained in three independent experiments. (**D**) Deconvolved wide-field fluorescence microscopy imaging of EGFP-tagged late endosomal/lysosomal marker RAB7A after 24 h SB202190 treatment. Scale bar: 10 µm. The diameter of RAB7-positive structures was determined, and the number of vacuoles larger than 1 μm per cell was plotted in the graph. The experiment was performed three times with similar results. (**E**) Effect on vacuolization was visualized by bright-field images in cells treated for 20 h with SB202190 alone and in combination with sucrose (0.5 M) or EIPA (50 μM). Scale bar: 50 µm. The graph shows the number of vacuoles per cell, quantified using ImageJ/Fiji (find maxima—bright spots above a certain threshold). Dying rounded cells were excluded from the analysis. The experiment was repeated three times with a similar response to the addition of sucrose and EIPA.

The above results indicated the possibility that SB202190-induced vacuole-like structures could form in response to a disruption of vesicular endocytic transport, a molecular pathway that is responsible for the active transport of membrane proteins, including receptors regulating growth signaling, and for the uptake of vital nutrients from the extracellular environment [38]. Small molecule fluorescent dyes such as acridine orange or LysoTracker Green DND-26 stain acidic compartments in living cells, including endosomes and lysosomes. Both dyes readily marked the SB202190-induced vacuoles in A375 melanoma cells, supporting the possibility of their endolysosomal origin (Figure 3A,B). Using confocal fluorescence microscopy, we observed that only a small proportion of SB202190-induced vacuoles with a diameter larger than 1 µm express early endosomal marker RAB5 (Figure 3C). In contrast, most SB202190-induced vacuoles with a diameter larger than 1 µm express late endosome marker RAB7 at their surface (Figure 3D), indicating that the majority of enlarged vacuoles originated from the late endosome.

Moreover, sucrose-induced osmotic stress or the small molecule inhibitor 5-(N-Ethyl-N-isopropyl)amiloride (EIPA), which inhibits a form of endocytosis called macropinocytosis, prevented the formation of large vacuoles in SB202190-treated cells (Figure 3E). The negative impact of EIPA on RAB7-positive vacuole-like vesicles was also observed using fluorescence microscopy (Figure S5). These data further supported the endocytic origin of the enlarged vesicles. Furthermore, live-cell imaging indicated that most SB202190-induced vacuoles could not be recycled back to the plasma membrane, suggesting a block in later stages of endosomal trafficking (Video S1).

### 2.3. Pyridinyl Imidazole Compounds Partly Mimic PIKfyve Inhibition in Melanoma Cells

**Figure 4:**
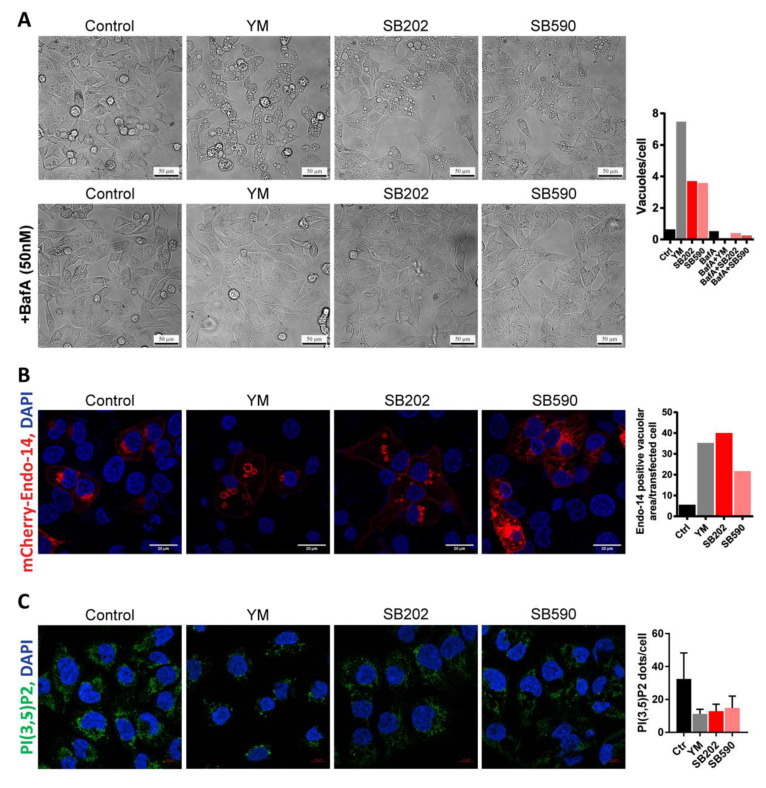
Phenotype induced in melanoma cells by pyridinyl imidazole compounds partly mimics PIKfyve inhibition. A375 cells were treated 24 h with pyridinyl imidazole inhibitors SB202190 (SB202; 15 μM), SB590885 (SB590; 5 µM), and PIKfyve inhibitor YM201636 (YM; 1 µM). (**A**) A phase-contrast analysis of the vacuolization induced by the inhibitors and the effect of the cotreatment with bafilomycin A (BafA; 50 nM). Scale bar: 50 µm. Vacuoles per cell were quantified using ImageJ/Fiji (find maxima—bright spots above a certain threshold) and presented in the graph. Dying rounded cells were excluded from the analysis. Three independent experiments showed a similar effect of the addition of BafA. (**B**) Confocal microscopy analysis of fluorescent protein-labeled endosomal marker (mCherry-Endo-14). Scale bar: 20 µm. The area of mCherry-positive vacuolar structures was quantified in each treatment and plotted in the graph. Three independent experiments showed similar results. (**C**) Confocal fluorescence microscopy detection of endogenous phosphatidylinositol 3,5-bisphosphate (PI(3,5)P2). Scale bar: 10 µm. Three images of different parts of the same specimen were acquired and analyzed using ImageJ/Fiji. Images were thresholded by the signal intensity, and the amount of PI(3,5)P2 foci per cell was determined. Similar results were obtained in two independent experiments.

The apparent defect in the endocytic pathway promoting a marked increase in the volume of endolysosomes led us to search the literature for a similar phenotype. Inhibition of the FYVE finger-containing phosphoinositide kinase (PIKfyve) had been previously reported to induce extensive cytoplasmic vacuolization in some cell types [39,40]. In A375 melanoma cells, the phenotype induced by a small-molecule PIKfyve inhibitor YM201636 was strikingly similar to that induced by SB202190, including the characteristic accumulation of large vesicles (Figure 4A). As the BRAF inhibitor SB590885 bears significant structural similarity to p38 MAPK inhibitors SB202190 and SB203580, we hypothesized that it might also be capable of targeting endolysosomal trafficking. Indeed, we also observed the accumulation of large vacuole-like vesicles in SB590885-treated A375 melanoma cells (Figure 4A). Furthermore, a small molecule inhibitor of vacuolar H+ ATPase bafilomycin A1 that can prevent the formation of vacuoles in response to PIKfyve inhibition [41] also blocked the vacuolization induced by pyridinyl imidazole compounds (Figure 4A).

Next, we ectopically expressed the mCherry-tagged endosomal marker RhoB (mCherry-Endo-14) in A375 melanoma cells. Using confocal fluorescence microscopy, we found that it localized to the membranes of vesicles induced by the inhibitor of PIKfyve as well as vesicles induced by the pyridinyl imidazole compounds (Figure 4B). PIKfyve is responsible for the synthesis of phosphatidylinositol 3,5-bisphosphate (PI(3,5)P2) [42], and this lipid appears to be critical for the proper maturation of endosomes [43]. To our surprise, the cellular staining patterns detected by immunofluorescence with an anti-PI(3,5)P2 antibody in YM201636- and pyridinyl imidazole-treated A375 cells were very similar (Figure 4C). Collectively, these results suggested that the pyridinyl imidazole compounds and the PIKfyve inhibitor YM201636 caused a very similar defect in endocytosis, potentially by targeting similar signaling pathways. Nevertheless, whether these changes might impact on the growth and survival of cancer cells remained unclear.

### 2.4. Pyridinyl Imidazole Compounds Induce Changes in mTOR Subcellular Localization in A375 Melanoma Cells

Bridges et al. reported that Raptor, an essential subunit of mTORC1, could interact with PI(3,5)P2, and PIKfyve was necessary for the subcellular localization and activation of mTORC1 in 3T3-L1 adipocytes [40]. The activation of mTORC1 requires the translocation of the mTOR kinase to the lysosomal surface [22]. The process involves interactions between lysosomal v-ATPase and a pentameric protein complex called Ragulator [44]. This complex possesses a guanine nucleotide exchange factor (GEF) activity towards the Rag GTPases that can recruit mTORC1 for activation at the lysosomal surface [19,45,46].

**Figure 5:**
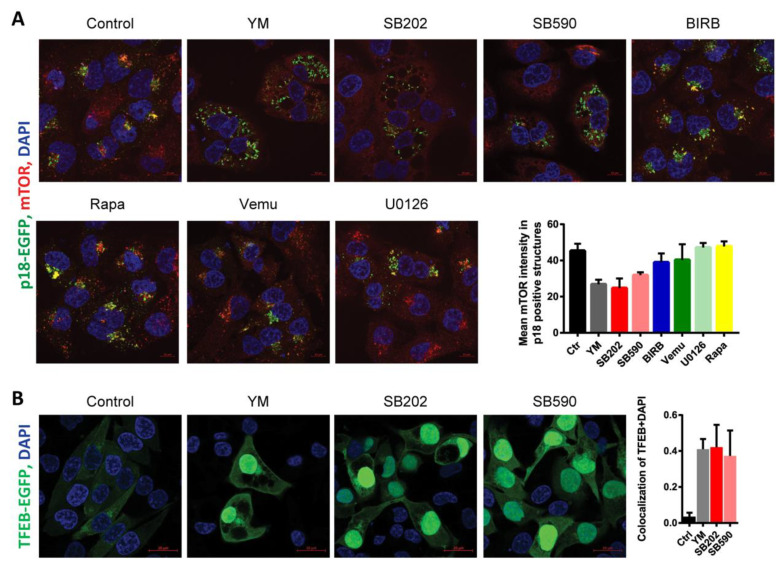
Pyridinyl imidazole compounds induce the delocalization of mechanistic target of rapamycin (mTOR) kinase from lysosomes in melanoma cells. (**A**) EGFP-labeled p18/LAMTOR1, endogenous mTOR, and nuclei (DAPI) were visualized by confocal fluorescence microscopy in A375 cells treated with YM201636 (YM; 1 μM), SB202190 (SB202; 15 μM), SB590885 (SB590; 5 μM), BIRB796 (BIRB; 15 µM), Vemurafenib (Vemu; 1 μM), U0126 (10 μM), and Rapamycin (Rapa; 200 nM). Scale bar: 10 µm. The co-occurrence of two fluorescent signals (p18/LAMTOR1-EGFP and endogenous mTOR) was quantified using ImageJ/Fiji. Measurements of three pictures from the same experiment were used for analysis. Three independent experiments showed similar results. (**B**) A375 cells were transiently transfected with a plasmid construct encoding EGFP-tagged transcription factor EB (TFEB) and treated for 24 h with YM201636 (YM; 1 μM), SB202190 (SB202; 15 μM), and SB590885 (SB590; 5 μM) and the nuclear translocation of TFEB was analyzed using confocal fluorescence microscopy. Scale bar: 20 µm. Colocalization of TFEB and DAPI was quantified using ImageJ/Fiji software (Pearson’s correlation coefficient), and the results were plotted in a graph. Similar results were obtained in three independent experiments.

We hypothesized that changes induced in the endolysosomal compartment of melanoma cells in response to PIKfyve inhibition and pyridinyl imidazole compounds might interfere with mTOR subcellular localization. To study this possibility, we expressed in A375 cells EGFP-tagged lysosomal Ragulator complex protein p18/LAMTOR1 and analyzed its colocalization with endogenous mTOR kinase using fluorescence microscopy. Protein p18/LAMTOR1 serves as a scaffold for the assembly of the Ragulator–Rag GTPase complex and is responsible for its anchoring to the lysosomal membrane [47]. The mTOR-p18/LAMTOR1 colocalization pattern was also analyzed in cells treated with drugs targeting the mTORC1 kinase (Rapamycin), p38 MAPK (BIRB-796), and ERK MAPK signaling (U0126, Vemurafenib). Even though the A375 melanoma cells were grown in full media, the PIKfyve inhibitor, as well as the pyridinyl imidazole compounds SB202190 and SB590885, all seemed to disrupt lysosomal mTOR targeting as the mTOR staining pattern changed from dot-like structures to diffuse cytoplasmic staining (Figure 5A). Importantly, a switch to mTOR diffuse staining pattern upon the extended treatment was not observed in response to the p38 MAPK inhibitor BIRB796, the MEK kinase inhibitor U0126, or the BRAF inhibitor vemurafenib. The results indicated that the observed effect of pyridinyl imidazole compounds was likely not linked to their capacity to inhibit p38 and ERK MAPK signaling pathways (Figure 5A). Interestingly, the results of the confocal microscopy suggested that p18/LAMTOR1 might not be evenly distributed on the surface of enlarged endolysosomes induced by pyridinyl imidazoles. The Ragulator complex protein appeared to form clusters on the surface of the large vacuole like structures (Figure 5A). A detailed time-lapse microscopy analysis indicated that p18/LAMTOR1 might preferentially cluster at the interface of the large vacuoles (Video S2).

The mTORC1 activity directly regulates the subcellular localization of the transcription factor EB (TFEB), a master regulator of lysosomal biogenesis [48]. Starvation, disruption of lysosomal function, and pharmacological inhibition of mTORC1 can stimulate TFEB-dependent transcription by promoting TFEB nuclear translocation [49,50]. Interestingly, PIKfyve inhibition was also shown to induce nuclear accumulation of TFEB [51,52]. In our experiments, the PIKfyve inhibitor YM201636 and pyridinyl imidazole inhibitors SB202190 and SB590885 all promoted nuclear localization of TFEB (Figure 5B), indicating that the drug-induced disruption of mTOR lysosomal tethering could inhibit mTORC1 activity in melanoma cells.

The recruitment of mTOR complex 1 to the lysosome is strictly regulated by the availability of nutrients, in particular the cellular levels of glutamine, arginine, methionine, and leucine [22,25,53,54]. Oncogenic mutations in the ERK pathway can promote macropinocytosis, stimulating cellular uptake of proteins, amino acids, and other nutrients from the environment, enhancing amino acid-dependent mTORC1 activation and cancer cell growth [55,56]. The results presented in Figure 3E indicate that pyridinyl imidazole compounds might modulate macropinocytosis in BRAF-mutated melanoma cells. To test the potential impact of pyridinyl imidazole treatment on nutrient uptake and processing via macropinocytosis in melanoma cells, we analyzed the cellular uptake of DQ-BSA using flow cytometry. DQ-BSA is a self-quenched fluorescently labeled albumin that emits fluorescence upon lysosomal degradation [57] and is commonly used as a marker for macropinocytic flux [55,58]. We compared DQ-BSA fluorescence in A375 cells treated with DMSO (control) and SB202190. We also used YM201636 because PIKfyve inhibition was shown to profoundly reduce the cellular levels of DQ-BSA fluorescence [59]. The total amount of DQ-BSA uptake/fluorescence was significantly decreased by both SB202190 and YM201636 (Figure S6A), suggesting that either macropinocytosis or the subsequent lysosomal proteolytic degradation of the cargo could be affected by the pyridinyl imidazole treatment. To find out if pyridinyl imidazoles and PIKfyve inhibitors could modulate subcellular mTORC1 localization by limiting the availability of essential nutrients, we used nuclear magnetic resonance (NMR) spectroscopy to analyze the cellular levels of several critical amino acids in inhibitor-treated A375 cells. Surprisingly, even though PIKfyve inhibition and pyridinyl imidazole compounds induced similar changes in the endocytic pathway and both inhibited mTOR localization to lysosomes, the results suggested a different trend in changes in cellular levels of free amino acids in cells treated with YM201636 in comparison to SB202190 and SB590885 (Figure S6B). These data indicated that changes in the lysosome properties, rather than an acute shortage of nutrients, could be the cause for the observed mTOR delocalization from lysosomes in A375 melanoma cells.

### 2.5. Pyridinyl Imidazole Compounds Induce Dynamic Changes in mTORC1 Activity in Melanoma Cells

**Figure 6:**
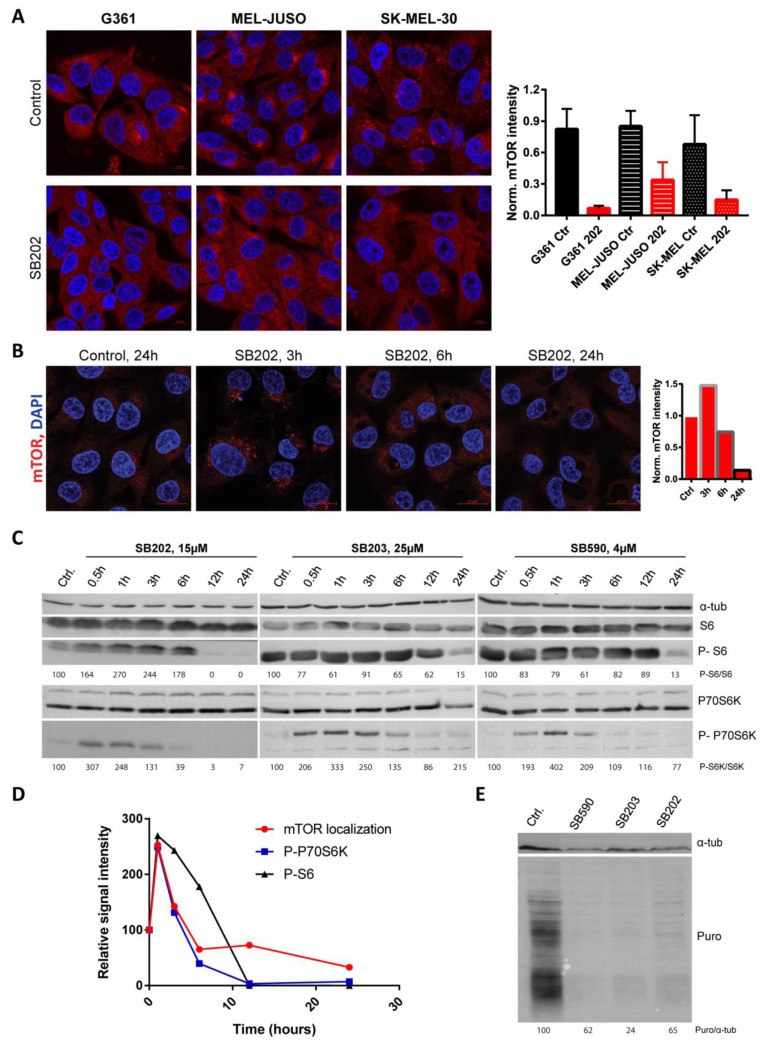
Pyridinyl imidazole compounds promote dynamic changes in mTOR activity in human melanoma cells. (**A**) BRAF-mutated G361 and NRAS-mutated MEL-JUSO and SK-MEL-30 human melanoma cells were stained for endogenous mTOR after 24 h treatment with SB202190 (SB202; 15 µM). Images were thresholded by a constant value of mTOR intensity, independently for each cell line, and mean mTOR intensity was quantified for three images per condition using ImageJ/Fiji. Scale bar: 10 µm. (**B**) Localization of endogenous mTOR in BRAF-mutated A375 melanoma cells treated for 0, 3, 12, and 24 h with SB202190 (SB202; 15 μM). Scale bar: 20 µm. The changes in mTOR signal intensity were quantified using ImageJ/Fiji and plotted in the graph as relative values. (**C**) Time-dependent changes in the activity of mTOR downstream target p70 S6K and phosphorylation of ribosomal S6 protein were analyzed by Western blotting. A375 cells were treated with pyridinyl imidazole compounds SB202190 (SB202), SB203580 (SB203), and SB590885 (SB590). (**D**) Graph illustrating changes in mTOR localization (Figure 6B) and activity (Figure 6C) at various time points upon SB202190 exposure. (**E**) Puromycylation assay in A375 cells treated with SB202190 (SB202), SB203580 (SB203), and SB590885 (SB590). Newly synthesized polypeptides were pulse-labeled with puromycin (Puro) and analyzed by Western blot. Tubulin alpha (α-tub) served as a loading control. The ratio between puromycin and tubulin signal intensity was determined using ImageJ/Fiji.

Before analyzing the impact of pyridinyl imidazole-induced mTOR delocalization from lysosomes on the physiology of melanoma in more detail, we wanted to make sure that the compounds’ effect was not limited to A375 melanoma. We, therefore, analyzed the mTOR response using immunofluorescence in a different human melanoma cell line bearing the BRAF V600E mutation (G361). In response to 24 h treatment with SB202190, the mTOR kinase staining pattern became diffuse (Figure 6A), similar to what we observed in A375 cells in previous experiments. Importantly, a very similar shift in the mTOR subcellular localization was also observed in two human melanoma cell lines expressing mutated NRAS, MEL-JUSO and SK-MEL-30 (Figure 6A). These data indicated that the observed effect of pyridinyl imidazole compounds on the mTOR signaling in human melanoma did not depend on the presence of the BRAF V600E activating mutation in the ERK signaling pathway. Three independent experiments showed similar results.

To further characterize the impact of pyridinyl imidazole-induced changes in the endolysosomal pathway on mTOR localization and activity, we performed immunofluorescence analysis of the subcellular localization of mTOR in SB202190-treated A375 cells at various time points. The mTOR kinase still localized to the surface of SB202190-induced vesicles at shorter time points (3 and 6 h), while exhibiting mostly diffuse cytoplasmic staining pattern with no apparent accumulation on the surface of the vacuoles at 24 h (Figure 6B). Interestingly, the quantification of mTOR signal intensity in fluorescent images indicated an increase in lysosomal mTOR targeting in response to three-hour treatment with SB202190, but this effect was reverted in longer treatments (Figure 6B). Three independent experiments showed similar results.

Next, we analyzed the potential impact of these dynamic changes of mTOR subcellular localization on mTORC1 activity in melanoma cells. Using Western blotting, we determined the phosphorylation status of downstream mTORC1 targets ribosomal S6 kinase (p70 S6K) and ribosomal protein S6 (RPS6). Interestingly, we found that shorter treatments of up to 3 h with pyridinyl imidazole compounds SB202190, SB203580, and SB590885 generally promoted the activity of p70 S6K and, in some cases, we also detected increased phosphorylation of its target RPS6 (Figure 6C). These results indicated that the compounds had a positive effect on mTORC1 activity in A375 cells. However, more extended treatments (12 and 24 h) caused a drop in p70 S6K and RPS6 phosphorylation, suggesting that the drug-induced increase in mTORC1 activity was only transient and the mTOR signaling was inhibited at later time points (Figure 6C). Three independent experiments showed similar results (additional Western blots are available in Figure S1). A subsequent analysis of the data revealed that the dynamics of changes in mTORC1 activity in response to SB202190, to some extent, correlated with its localization pattern (Figure 6D). 

Taking into account the vital role of mTOR in the regulation of translation, we expected that inhibition of mTORC1 activity and RPS6 phosphorylation observed in melanoma cells in response to longer treatments might negatively affect the rates of protein synthesis [60]. To test this effect, we used puromycin to pulse label newly synthesized polypeptides in A375 melanoma cells treated with DMSO (negative control), SB202190, SB203580, and SB590885. The results revealed that the rate of translation was downregulated by all tested pyridinyl imidazole compounds (Figure 6E), implying that targeting mTORC1 signaling with pyridinyl imidazole drugs structurally related to SB202190 could have a substantial impact on the physiology of melanoma cells. Three independent experiments showed similar results (additional Western blots are available in Figure S1).

### 2.6. Pyridinyl Imidazole Drugs Sensitize A375 Melanoma Cells to ER Stress

**Figure 7:**
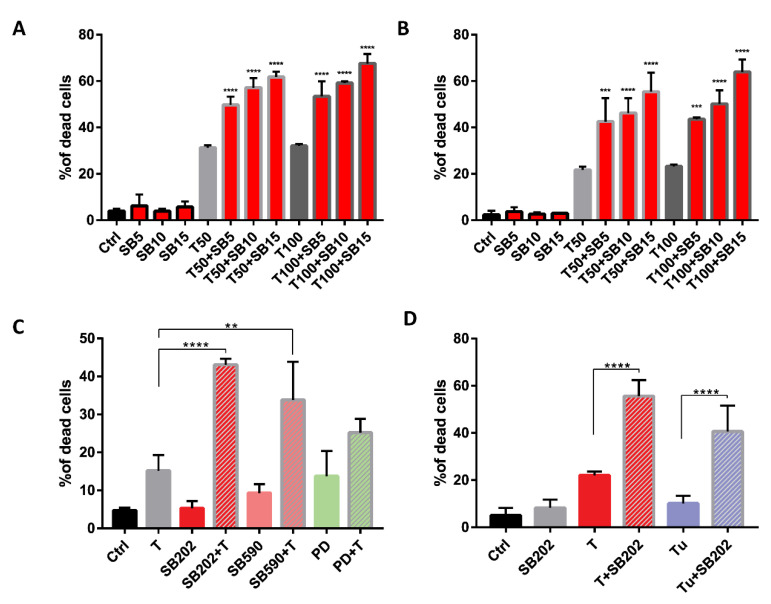
Pyridinyl imidazole compounds sensitize human melanoma cells to endoplasmic reticulum (ER) stress. Propidium iodide-based flow cytometry analysis of cell viability treated with small molecule compounds for 48 h. Presented data were obtained in three independent experiments. (**A**) Viability of BRAF-mutated A375 cells treated with SB202190 (SB; 5, 10, and 15 μM) in combination with ER stressor thapsigargin (T; 50 and 100 nM). (**B**) Viability of NRAS-mutated MEL-JUSO melanoma cells treated with SB202190 (SB; 5, 10, and 15 μM) in combination with thapsigargin (T; 50 and 100 nM). (**C**) Viability of A375 cells treated with thapsigargin (T; 50 nM) together with pyridinyl imidazole inhibitors SB202190 (SB202; 15 μM) and SB590885 (SB590; 5 μM), and MEK inhibitor PD184352 (PD; 0.5 μM). (**D**) Viability of A375 cells treated with SB202190 (SB202; 15 μM) in combination with ER stress inducers thapsigargin (T; 50 nM) and tunicamycin (Tu; 0.5 μM). ** denotes *p* < 0.01, **** indicates *p* < 0.0001.

Our data suggested that pyridinyl imidazole compounds disrupt the normal function of the endolysosomal pathway in melanoma cells. Such a disruption could have a negative impact on the autophagic flux, which was reported to be essential for the survival of cells subjected to endoplasmic reticulum (ER) stress [61]. We, therefore, hypothesized that pyridinyl imidazole compounds could sensitize cancer cells to the disruption of endoplasmic reticulum function. To test this possibility, we treated A375 cells with SB202190 alone and in combination with ER stress inducer thapsigargin for 48 h and analyzed cell viability using flow cytometry. The results presented in Figure 7A suggested a significant negative impact of SB202190 on the ability of BRAF-mutated melanoma cells to cope with ER stress. Next, we wanted to experimentally confirm that the effect of the tested drug combinations on melanoma cell viability could not be linked to the ability of SB202190 to inhibit the ERK pathway in A375 cells. Therefore, the same experiment was performed in the NRAS-mutated melanoma cell line MEL-JUSO, in which SB202190 did not block the ERK signaling. The results presented in Figure 7B are very similar to the results obtained in A375 cells, suggesting that the enhanced cytotoxicity of the SB202190-thapsigargin drug combinations was not limited to BRAF-mutant cells, and thus did not depend on the type of oncogenic mutation driving the ERK MAPK pathway in melanoma cells. A sharp increase in thapsigargin toxicity when combined with pyridinyl imidazole compounds was also observed in A375 melanoma cells treated with SB590885, but not when thapsigargin was combined with the MEK inhibitor PD184352 (Figure 7C). Last, but not least, the potent synergistic cytotoxicity towards melanoma cells was also observed when we combined SB202190 with another ER stressor tunicamycin, confirming that pyridinyl imidazole compounds significantly enhanced the cellular toxicity of ER stressors (Figure 7D).

## 3. Discussion

The BRAF oncogenic signaling is a prime therapeutic target in melanoma, and small molecule BRAF inhibitors dabrafenib and vemurafenib have been approved for clinical use. While they are extending the survival of melanoma patients, there is still room for improvement. BRAF-targeted therapy is commonly associated with acquired drug resistance and undesired off-target effects, such as the paradoxical activation of RAF signaling in healthy tissue [6,62].

Another approach to prevent acquired drug resistance could be a simultaneous targeting of two essential signaling pathways in melanoma cells. Reports are suggesting that overactivation of the PI3K/AKT/mTOR pathway might contribute to the survival of melanoma cells upon BRAF V600E inhibition and that dual targeting of advanced human cancers with inhibitors of the PI3K/AKT/mTOR and RAF/MEK/ERK pathways might be beneficial [7,63]. A close link between these two signaling pathways in BRAF mutated melanoma cells was also shown in a study that analyzed intrinsic cross-resistance to inhibitors of the two pathways [64]. 

The mTOR kinase is a central hub for the regulation of cell growth and proliferation, integrating the inputs from signaling pathways stimulated by growth factors, cell energy metabolism, and the availability of essential nutrients. The mTOR kinase activity can also respond to various stress stimuli, for example, hypoxia, ER stress, or oxidative stress, and contribute to the adaptation of cells to stress [65,66].

In the current paper, we show that the pyridinyl imidazole inhibitors SB202190 and SB203580, widely used as chemical probes to study the p38 MAPK signaling, also simultaneously target the BRAF V600E oncogene-driven ERK signaling and the mTORC1 growth signaling pathway in human melanoma cells. Importantly, a structurally related first-generation BRAF inhibitor SB590885 also disrupted mTORC1 signaling, indicating that the dual targeting of BRAF and mTORC1 could be a general feature of pyridinyl imidazole compounds structurally related to SB202190. We present data suggesting that the inhibition of mTORC1 by pyridinyl imidazole drugs is ERK-independent. It could be linked to the disruption of the endocytic pathway and induction of cellular vacuolization, mimicking the response of melanoma cells to the inhibition of PIKfyve, a lipid kinase that catalyzes the synthesis of PI(3,5)P2 from PI3P [42]. PIKfyve operates through cationic transporter TRPML1 (transient receptor potential mucolipin 1) to regulate the vacuolar size and promote nutrient recovery under starvation [43]. Interestingly, the fusion of amphisomes with lysosomes was impaired in Drosophila Trpml−/− cells, leading to a decrease in TORC1 signaling [67]. Dong and colleagues showed that PI(3,5)P2 could directly bind the TRPML1 channel, and this interaction modulates endosomal trafficking and the activity of endolysosomes [68]. It is also of interest that Raptor, an essential component of the mTORC1 complex, which plays a vital role in determining its subcellular localization [22], was also shown to directly interact with PI(3,5)P2 [40].

We show that the pyridinyl imidazole compounds induce delocalization of mTOR from the lysosome, possibly owing to the changes of the lysosomal membrane, promoting enhanced endolysosomal fusion and decreased fission [69]. The observed sequestration of the Ragulator complex protein p18/LAMTOR1 at the interface of the large vesicles could also contribute to further cellular vacuolization as p18/LAMTOR1 was shown to be crucial for late endosome–lysosome fusion. Its loss caused the accumulation of endosomes with abnormal sizes [70] and lower DQ-BSA uptake [71].

The dual targeting of BRAF and mTOR signaling pathways, which are essential for the growth of a significant proportion of melanomas, suggested that pyridinyl imidazole compounds could have a therapeutic potential in BRAF-mutated melanoma. While only melanoma cells bearing the BRAF V600E mutation responded in our assays to pyridinyl imidazole compounds by ERK signaling inhibition, the cellular vacuolization, and delocalization of mTOR kinase was observed in both NRAS- and BRAF-mutant cells. We hypothesized that the strong disruptive effects of these molecules on the endolysosomal compartment and mTOR signaling might also create additional vulnerabilities in cancer cells independent of their MAPK pathway mutational status.

A recent report showed that translocation of the entire ERK signaling pathway to endoplasmic reticulum could drive therapy resistance in BRAF-mutant melanoma [72], indicating a therapeutic potential of simultaneous targeting of ER and BRAF in melanoma. We, therefore, hypothesized that pyridinyl imidazole compounds might disrupt the bidirectional crosstalk between the endolysosomal compartment and the endoplasmic reticulum that is required for the adaptation of cells to ER stress [73]. In response to ER malfunction, the unfolded protein response (UPR) is initiated by three sensors located in the ER membrane: double-stranded RNA-activated protein kinase (PKR)-like ER kinase (PERK), activating transcription factor 6 (ATF6), and inositol-requiring enzyme 1 (IRE1). All three stimulate pathways that can contribute to the induction of macroautophagy (autophagy) [74,75]. Autophagy is a coordinated process involving the sequestration of parts of the cytoplasm, protein aggregates, and damaged organelles into autophagosomes, double-membrane endosome-like vesicles, which fuse with lysosomes to form autolysosomes [76]. Interestingly, the levels of PI(3,5)P2 can also modulate autophagy. The lipid phosphatase INPP5E (inositol polyphosphate-5-phosphatase E), which decreases PI(3,5)P2 levels on lysosomes, was identified as a novel regulator of autophagy essential for autophagosome-lysosome fusion [77].

The mTORC1 complex is a negative regulator of autophagy that can directly modulate the activity of critical players in autophagy induction, for example, ULK1, ATG13, and ATG14. Moreover, active mTORC1 kinase can phosphorylate the transcription factor TFEB and a closely related protein TFE3, essential regulators of lysosomal biogenesis, and prevent their nuclear translocation [78,79]. Interestingly, the nuclear translocation of TFEB and TFE3 can also proceed in an mTORC1-independent, but PERK-dependent manner in response to ER stress [80]. In any case, the upregulation of autophagy can help cells to adapt and survive under the ER stress conditions [61,81]. Importantly, both melanoma subtypes responded in our experiments significantly stronger to the combination of SB202190 with the ER stressor thapsigargin than to individual compounds, suggesting that pyridinyl imidazole compounds could have a negative effect on the autophagy-mediated adaptation of melanoma cells to ER stress.

## 4. Materials and Methods 

### 4.1. Cell Culture and Treatments

Human melanoma cell lines A375, G361, COLO-800, MEL-JUSO, SK-MEL-30, and IPC-298 were purchased from the European Collection of Cell Cultures (ECACC; Salisbury, UK). All cell lines were maintained at 37 °C in a humidified atmosphere containing 5% CO2. IPC-298, MEL-JUSO, COLO-800, and A375 cells were cultured in RPMI-1640 (Sigma-Aldrich, Prague, Czech Republic). G361 cells were propagated in McCoy′s 5a (Thermo Fisher Scientific, Prague, Czech Republic) and SK-MEL-30 in Dulbecco’s modified Eagle’s medium (Thermo Fisher Scientific). Growth media were supplemented with 10% fetal bovine serum (FBS), 2 mM L-glutamine, penicillin (100 IU/mL), and streptomycin (100 μg/mL). The reporter cell line for measuring ERK pathway activity was prepared by stable transfection of A375 cells with pKrox24(MapErk)Luc plasmid construct [35]. Cells were regularly checked for mycoplasma contamination using Mycoplasma Detection Kit (Biotool, Munich, Germany) and DAPI staining followed by fluorescence microscopy. 

The following compounds were used for cell treatments: SB202190, SB239063, sucrose, EIPA, thapsigargin, and tunicamycin (Sigma-Aldrich); U0126 (Wako Chemicals, Neuss, Germany); SB203580, BIRB796, PD184352, YM201636, and rapamycin (Selleckchem, Munich, Germany); vemurafenib (Tinib-Tools, Olomouc, Czech Republic); SB590885 (MedChemExpress, Monmouth Junction, NJ, USA); and puromycin (Cayman Chemical, Ann Arbor, MI, USA). Stock solutions of the compounds were prepared in dimethyl sulfoxide (DMSO). Inhibitor stocks were diluted in pre-warmed cell culture medium and added to the cells. Controls received the corresponding amount of the vehicle.

### 4.2. Western Blotting

Cells were lysed in 2× Laemli sample buffer, and proteins in total cell lysates were separated by SDS-polyacrylamide gel electrophoresis (10 or 15% acrylamide) using mini vertical electrophoresis unit SE250 (Hoefer, Holliston, MA, USA). Proteins were transferred to polyvinylidene fluoride (PVDF) membranes (Merck Millipore, Prague, Czech Republic) in the Trans-Blot SD semi-dry transfer system (Bio-Rad, Prague, Czech Republic). Membranes were blocked with 5% non-fat milk in tris-buffered saline + 0.1% Tween 20 (TBST) for one hour at room temperature and incubated with primary antibodies overnight at 4 °C. The next day, membranes were washed 3 × 10 min in TBST and incubated with secondary antibodies for one hour at room temperature. Proteins of interest were visualized with enhanced chemiluminescence (ECL) substrate (Thermo Fisher Scientific) in the G:BOX detection system (Syngene, Cambridge, UK). The intensity of bands was quantified using ImageJ/Fiji. Original data are available in Figure S7.

Primary antibodies used for Western blot: mouse anti-α-tubulin (B-7; sc-5286), rabbit anti-pMEK1/2 (sc-7995), goat anti-MEK1 (C-18; sc-219), mouse anti-BRAF (F-7; sc-5284) (Santa Cruz Biotechnology), rabbit anti-p70 S6K (#2708), rabbit anti-phospho-p70 S6K (#9234), rabbit anti-phospho-S6 Ser235/236 (#4858), rabbit anti-S6 (#2217), rabbit anti-phospho-ERK1/2 T202/Y204 (#4370), rabbit anti-ERK1/2 (#9102) (Cell Signaling Technology, Danvers, MA, USA), mouse anti-Puromycin (MABE343) (Sigma-Aldrich), and mouse anti-PCNA (PC-10), kindly provided by Dr. Bořivoj Vojtěšek (Masaryk Memorial Cancer Institute, Brno, Czech Republic). Secondary antibodies conjugated to horseradish peroxidase (HRP): donkey anti-rabbit (sc-2357), anti-mouse (sc-516102), and anti-goat (sc-2020) (Santa Cruz Biotechnology, Heidelberg, Germany). All antibodies were used according to the manufacturers’ recommendations.

### 4.3. In Vitro Kinase Assays 

Melanoma cells (A375) were seeded at 10 cm plate, washed once with ice-cold phosphate-buffered saline (PBS), scraped out, and lysed as previously described [82,83]. The lysate was centrifugated at 13,000× *g* for 25 min at 4 °C. Mouse anti-BRAF (sc-5284; Santa Cruz Biotechnology) antibody was added (1 µg per each sample) to the lysate and incubated for 1–2 h at 4 °C on a slow rotator. Protein G Sepharose 4 Fast Flow beads (GE Healthcare, Chicago, IL, USA) were added and incubated for another 2–3 h at 4 °C on a slow rotator. Beads–immune complexes were washed as previously described [82,83]. The kinase assay was performed in the presence of 20 µM ATP and 500 ng of a recombinant human inactive MEK-1 as the substrate (Life Technologies, Prague, Czech Republic). A corresponding amount of DMSO (control) or SB202190 (5 µM) was added to the reaction performed for 40 min at 30 °C with gentle agitation. The kinase reactions were stopped by the addition of 20 µL 2 × SDS sample buffer, analyzed using Western blot, and density of bands was quantified via ImageJ/Fiji.

### 4.4. Transient Transfections and Fluorescence Microscopy

The day after seeding the cells to a glass coverslip, cells were transfected using TurboFect transfection reagent (Thermo Fisher Scientific) or FuGENE HD (Promega, Madison, WI, USA) following the manufacturer’s instructions. Twenty-four hours post transfections, cells were treated for the indicated time, fixed with 4% paraformaldehyde, permeabilized with 0.1% Triton X, and incubated with primary antibodies at 4 °C overnight. Secondary antibodies were used at room temperature for one hour. Then, coverslips were stained with DAPI (sc-3598; Santa Cruz Biotechnology) and washed three times with PBS. Images were acquired using an inverted confocal microscope Carl Zeiss LSM 700 (Jena, Germany) with Plan-Apochromat 63x/1.4 Oil DIC M27 objective (Zeiss) and processed in ZEISS ZEN Microscope Software or ImageJ/Fiji. Quantification was performed using at least three technical replicates to take into account the variability owing to differences in microscopy settings. All analyses were performed in at least two independent experiments.

Live cell imaging: Melanoma cells (A375) were seeded on MatTek 35 mm glass-bottom dishes (MatTek Corporation, Ashland, MA, USA), and the next day transfected using FuGENE HD (Promega) according to the manufacturer’s instructions. Cells were washed four hours later and treated with the inhibitor (SB202190, 15 μM) for 24 h. For imaging, the media was replaced with warm Live Cell Imaging buffer (Invitrogen) containing the inhibitor (SB202190, 15 μM) and supplemented with 20 mM glucose. Live cell imaging was performed on the Deltavision OMX V4 microscope (GE Healthcare) equipped with three water-cooled PCO.edge sCMOS cameras, a solid-state light source, and laser-based autofocus. Heated stage and an objective heater (20/20 Technologies, Wilmington, NC, USA) provided environmental control conditions. Images were deconvolved using softWoRx software and processed in ImageJ/Fiji [84,85]. 

Dyes used for cell staining: LysoTracker Green DND-26 (Thermo Fisher Scientific), Acridine Orange hemi(zinc chloride) salt (A6014, Sigma-Aldrich).

The following primary antibodies were used for immunofluorescence: rabbit anti-mTOR (#2983), rabbit anti-Rab5 (#3547; Cell Signaling Technology), mouse anti-PtdIns(3,5)P2 (Z-P035; Echelon Biosciences, Salt Lake City, UT, USA). Anti-rabbit Alexa Fluor 594/488 (A11012/A11008) and anti-mouse Alexa Fluor 488 (A11001) (Life Technologies) were used as secondary antibodies.

List of plasmids: mCherry-Endo-14 (Addgene, #55040), EGFP-Rab7A (Addgene, #28407), N1-p18-EGFP (Addgene, #42334), and EGFP-N1-TFEB (Addgene, #38119).

### 4.5. Quantitative Phase Imaging Analysis Using Coherence-Controlled Holographic Microscopy

Transmitted-light coherence-controlled holographic microscope (CCHM) was used to evaluate the distribution of the dry mass (DM) (e.g., proteins, carbohydrates, fats, amino acids, and nucleic acids) density in A375 cells and the drug-induced vacuoles. The microscope was designed and built at the Institute of Physical Engineering (IPE) and the Central European Institute of Technology (CEITEC), Brno University of Technology. The setup of the microscope and the principle behind the image reconstruction have previously been described in detail [86]. The CCHM quantitative phase images are formed by the phase shift between the object and reference wave detected using the interference of light. At every image point, the phase shift value is directly proportional to the cell dry-mass density [87].

For obtaining the images, we used 20×/ NA = 0.4 objectives, a digital CCD camera Astropix 1.4, and software developed at the IPE and CEITEC. The light source was a halogen lamp spectrally restricted by an interference filter (FWHM = 10 nm, maximum transmissivity at 650 nm). 

Melanoma cells (A375) were observed live in the imaging chamber with 5% CO2. Before imaging, the culture medium was replaced with an observation medium: Eagle’s minimal essential medium without phenol red (buffered to pH 7.4) (Sigma-Aldrich), supplemented with 10 % FBS and only one-third of the normal concentration of sodium bicarbonate.

### 4.6. Electron Microscopy

A375 melanoma cells were seeded on 35 mm plates, grown for 24 h and harvested 12 h after treatment, washed three times in 0.1 M cacodylate buffer (pH 6.98), and fixed for 2.5 h in 3% glutaraldehyde solution (prepared in 0.1 M cacodylate buffer containing 0.2 M saccharose). Afterward, post-fixation was performed in 1% osmium tetroxide in 0.1 M cacodylate buffer for 2.5 h at room temperature. Individual sections were stained with 2.5% uranyl acetate (6 min), lead citrate (3 min), and observed using transmission electron microscopy (Philips Morgagni, FEI Company, Eindhoven, The Netherlands).

### 4.7. Quantification of Intracellular Metabolites Using NMR Spectroscopy

Acetonitrile extraction was employed to quench cell metabolism and to extract low molecular weight compounds from A375 melanoma cells quantitatively [83,88]. Following removal of acetonitrile via vacuum concentration, dried extracts were resuspended in 550 μL of D_2_O (Sigma-Aldrich) containing 0.005% sodium 3-(trimethylsilyl)-propionate-2,2,3,3-d4 (TSP) (Sigma-Aldrich) used as both chemical shift reference and internal standard for metabolite quantification.

Information on the concentration of metabolites in individual samples was derived from volumes of corresponding signals in 1D 1H NMR spectrum. The assignment of signals in the NMR spectra of individual samples to a metabolite was achieved via a comparison of a sample spectrum with spectra of pure metabolites (Sigma-Aldrich). The 1D 1H spectra were measured at 700 MHz using a Bruker Avance III NMR spectrometer (Bruker, Billerica, MA, USA) equipped with a triple resonance room temperature probe using the zgpr pulse sequence (standard Bruker pulse program library). All spectra were acquired at 20 °C and processed using TopSpin 3.2 (Bruker). To make the comparison of metabolite concentration profiles among various samples possible, the signal intensities in individual samples were normalized to total protein concentration.

### 4.8. Flow Cytometry and MTT Proliferation Assay

For cell viability assay, melanoma cells were collected 48 h post-treatment and washed once with ice-cold PBS. Cell pellets were resuspended in ice-cold PBS, and 1 µg/mL propidium iodide (PI) (Sigma-Aldrich) was added to the suspension and cell fluorescence was measured using the Attune Acoustic Focusing Cytometer (Thermo Fisher Scientific). As live cells exclude PI, the percentage of dead cells was calculated based on the proportion of PI-positive cells in the population.

Uptake of BSA was measured in A375 melanoma cells, seeded in density 200,000 cells per well on a six-well plate. The next day, cells were treated with tested drugs for one hour, and then 10 ug/mL of DQ Red BSA (Thermo Fisher Scientific, D12051) was added to the medium for 30 min. Afterward, cells were chased for 75 min in DQ-BSA free medium (with inhibitors) and collected. Flow cytometry analysis was performed on LSR II flow cytometer (BD Biosciences, San Jose, CA, USA) using FACS Diva (BD Biosciences) software.

MTT assay was used to measure cellular metabolic activity as an indicator of cell viability and proliferation of melanoma cell lines (A375, G361, COLO-800, MEL-JUSO, SK-MEL-30, IPC-298). Cells were seeded at a density of 1–2 × 10^3^ cells/well in a 96-well plate and grown overnight. After treatment with inhibitors for 48 h, cells were incubated with 0.5 mg/mL MTT (3-(4,5-dimethylthiazol-2-yl)-2,5-diphenyltetrazolium bromide) for 4 h at 37 °C. Afterward, cells were centrifugated, and the water-insoluble formazan product was dissolved in DMSO (200 µL/well). The absorbance at 570 nm was determined using a microplate reader VersaMax (Molecular Devices, San Jose, CA, USA).

### 4.9. Statistical Analysis 

The analyses were performed using GraphPad Prism 7 (GraphPad Software, San Diego, CA, USA). Three or more independent experiments were performed for each data set, represented as mean + SD. Statistical analysis was done using Student’s t-test or analysis of variance (ANOVA) when multiple samples were compared. Values of * *p* < 0.05, ** *p* < 0.01, *** *p* < 0.001, and **** *p* < 0.0001 were considered statistically significant.

## 5. Conclusions

We identified pyridinyl imidazole compounds SB2020190, SB203580, and SB590885 as dual inhibitors of mutant BRAF kinase and mTOR signaling in melanoma cells. The dual targeting of essential pro-growth pathways in melanoma cells indicates the potential for the development of BRAF inhibitors based on the pyridinyl imidazole core that could be less prone to the development of acquired drug resistance. Moreover, the disruption of the endolysosomal compartment by pyridinyl imidazole drugs can sensitize melanoma cells to ER stressors, further underscoring their therapeutic potential.

## Supplementary Materials

The following are available online at https://www.mdpi.com/2072-6694/12/6/1516/s1, Figure S1: Additional independent replicates for all Western blots presented in this study; Figure S2: Pyridinyl imidazole compounds inhibit ERK signaling in A375 melanoma cells; Figure S3: Analysis of the proliferation of BRAF- and NRAS-mutated melanoma cell lines in the presence of pyridinyl imidazole compounds; Figure S4: Coherence-controlled holographic microscopy; Figure S5: Immunofluorescence microscopy; Figure S6. Nutrient uptake was negatively affected in A375 melanoma cells treated with pyridinyl imidazole compounds; Figure S7: Uncropped original Western blots; Video S1: SB202190 disrupts late endosomal trafficking in melanoma cells: Deconvolved wide-field live-cell fluorescence microscopy of A375 melanoma cells transiently transfected with a plasmid construct encoding EGFP-labeled late endosomal marker RAB7A (EGFP-Rab7A); Video S2: p18/LAMTOR1 clusters at contact sites of SB202190-induced vacuole-like vesicles: Live-cell imaging was performed using A375 cells transiently transfected with a plasmid construct encoding EGFP-labeled LAMTOR1 (N1-p18-EGFP).
