# Supplementary material for: Dual Targeting of BRAF and mTOR Signaling in Melanoma Cells with Pyridinyl Imidazole Compounds
Source: Cancers (Basel). 2020 Jun 10;12(6):1516. doi: 10.3390/cancers12061516 (PMC7352453; doi:10.3390/cancers12061516)

Two additional replicates of Fig. 1C

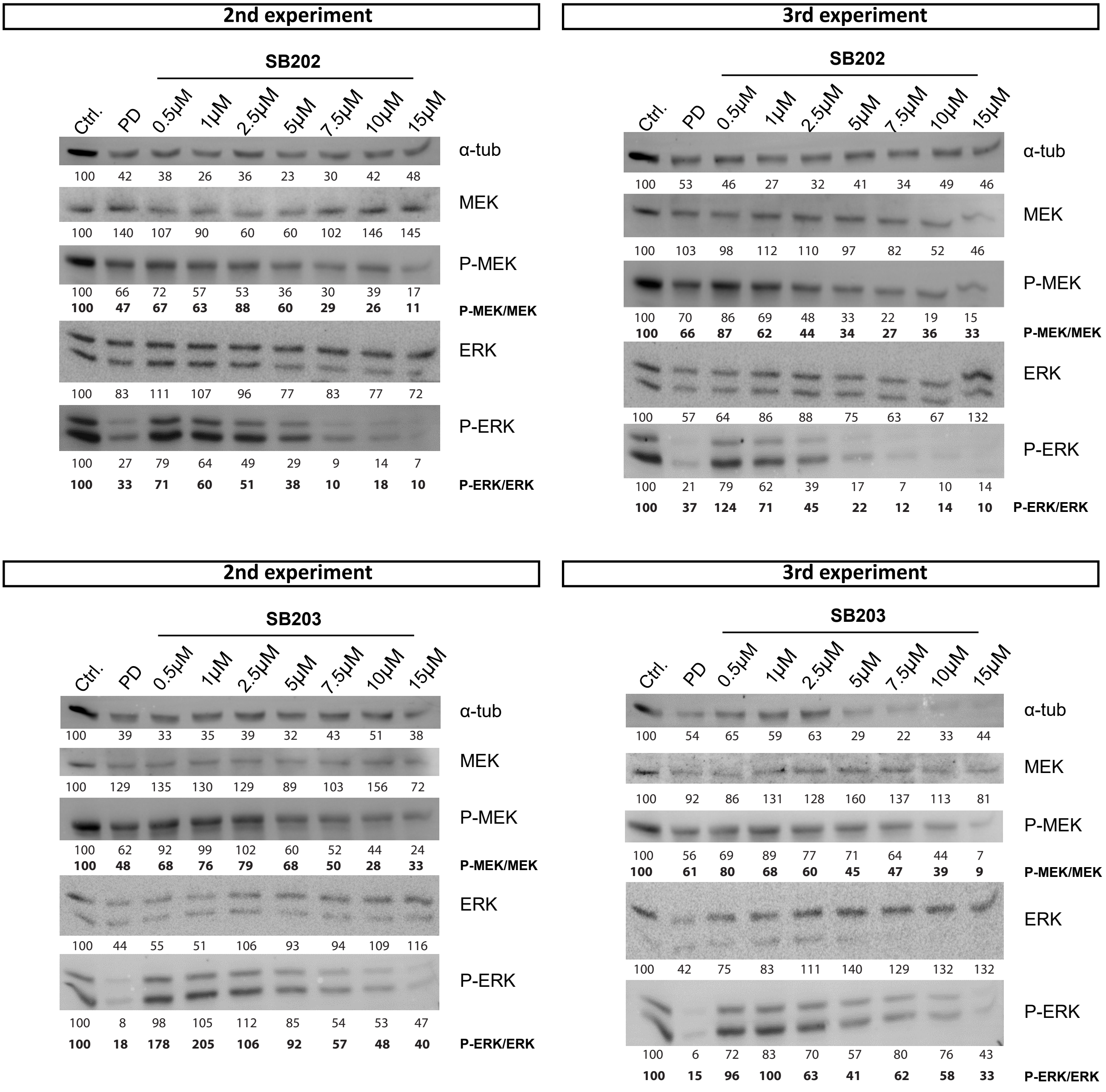

Two additional replicates of Fig. 1D

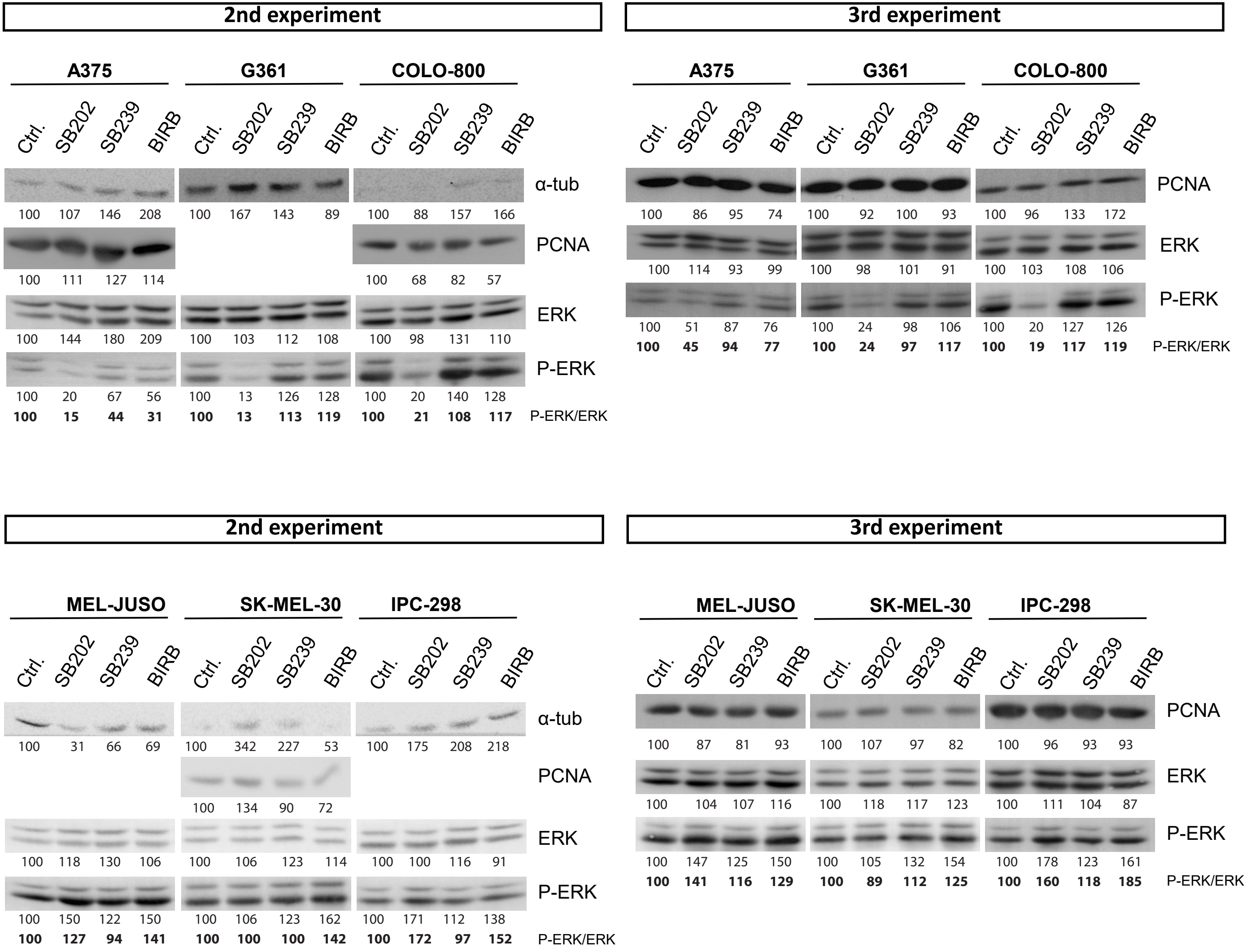

Two additional replicates of Fig. 6C

2nd experiment

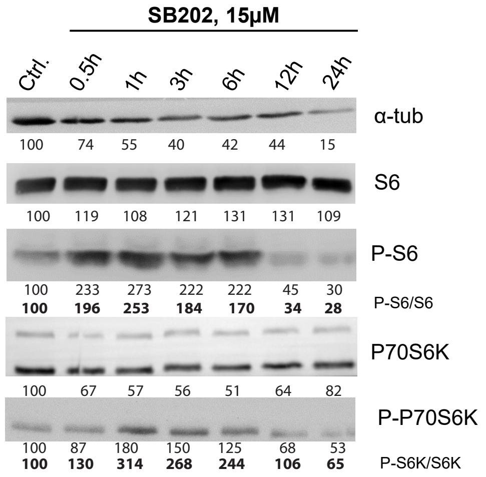

3rd experiment

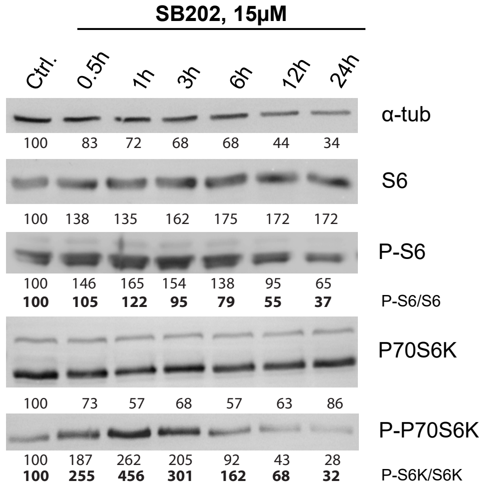

2nd experiment

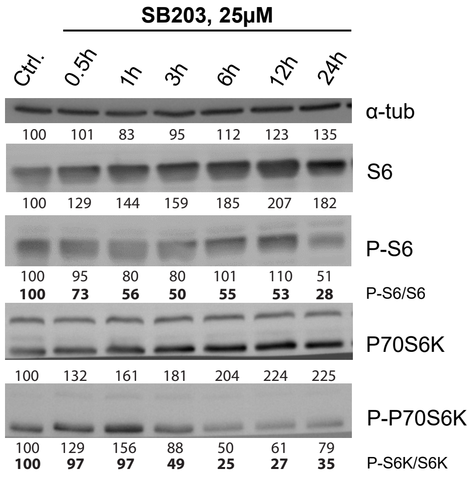

3rd experiment

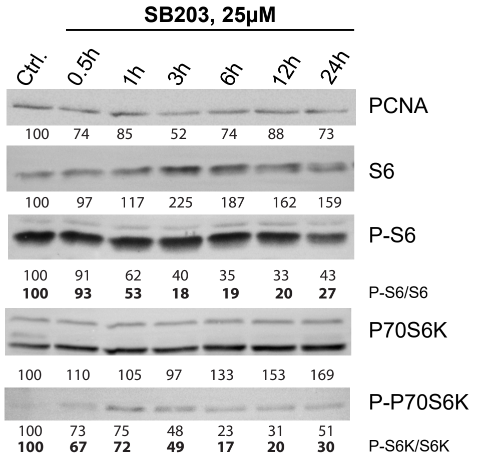

2nd experiment

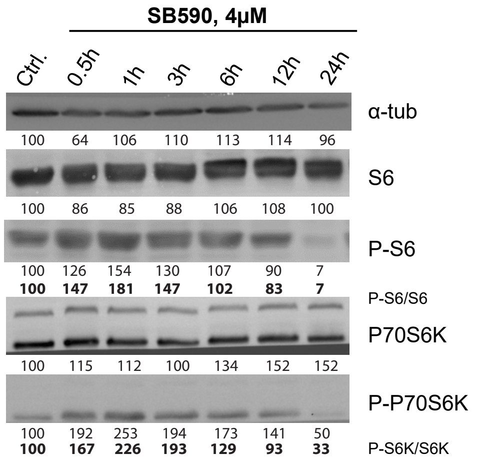

3rd experiment

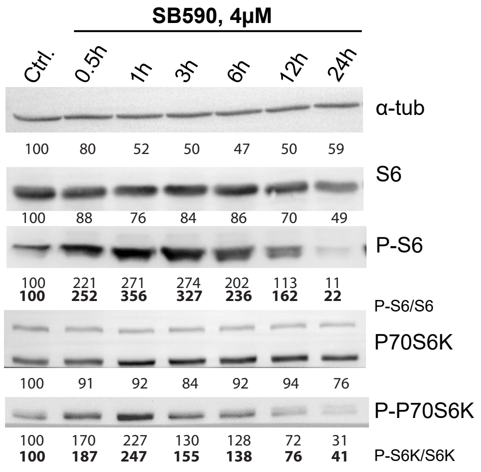

Two additional replicates of Fig. 6E

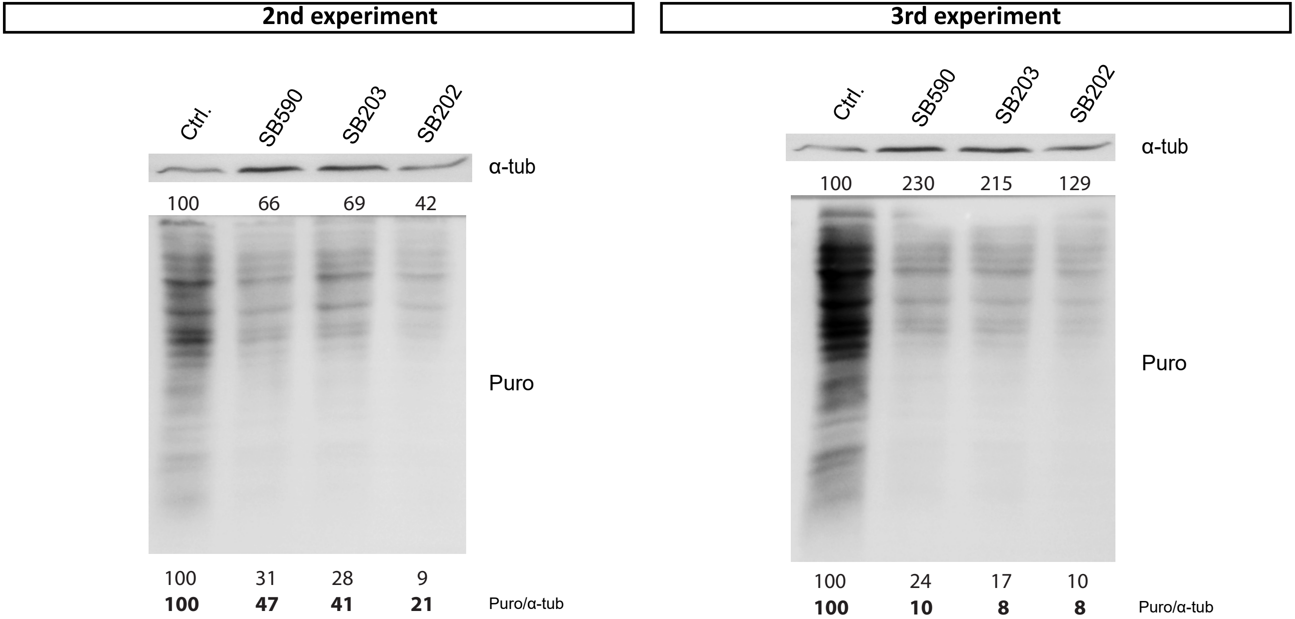

Supplement: Supplementary file 1 [file cancers-12-01516-s001.zip › Supplementary figure S1- additional WB.pdf]
