# Supplementary material for: Dual Targeting of BRAF and mTOR Signaling in Melanoma Cells with Pyridinyl Imidazole Compounds
Source: Cancers (Basel). 2020 Jun 10;12(6):1516. doi: 10.3390/cancers12061516 (PMC7352453; doi:10.3390/cancers12061516)
Supplement: Supplementary file 1 [file cancers-12-01516-s001.zip › Supplementary figure S2-S6.pdf]

Figure S2

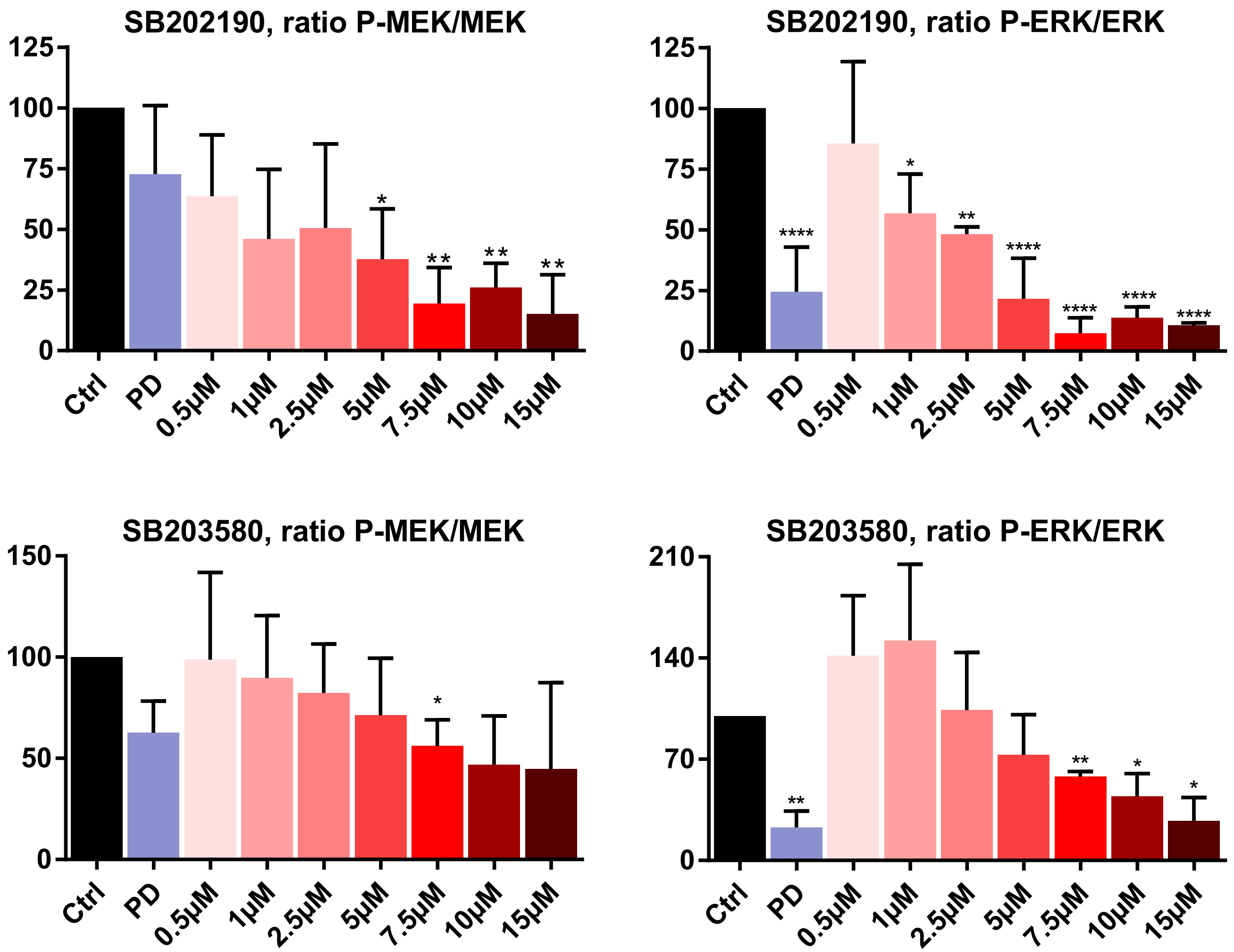

Supplementary figure S2. Pyridinyl imidazole compounds inhibit ERK signaling in A375 melanoma cells. Quantification of Western blot results obtained in three independent experiments (corresponding to the experiment presented in Figure 1C). A375 cells were treated for one hour with increasing concentrations of SB202190 and SB203580. MEK inhibitor PD184352 (PD; 100nM) was used as a positive control. Graphs show the relative ratio between phosphorylated proteins (P-MEK and P-ERK) and the total protein levels (MEK and ERK).

Figure S3

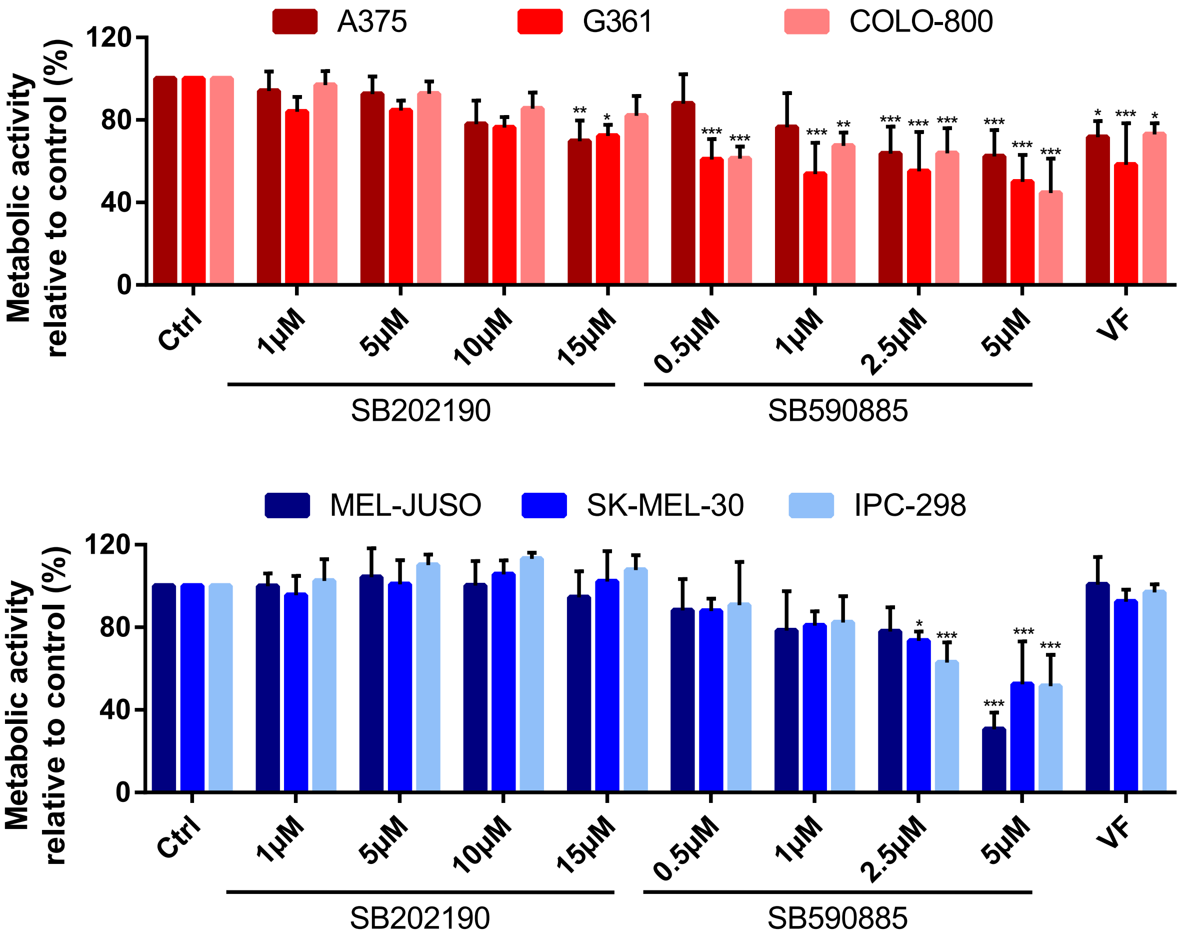

Supplementary figure S3. Analysis of the proliferation of BRAF- and NRAS-mutated melanoma cell lines in the presence of pyridinyl imidazole compounds. MTT assay was used to analyze the proliferation of six different melanoma cell lines (A375, G361, COLO-800, MEL-JUSO, SK-MEL-30, and IPC-298), exposed for 48 hours to different concentrations of SB202190 and SB590885, or a structurally unrelated BRAF inhibitor (Vemurafenib, VF; 1μM). The three independent experiments are presented in two graphs: BRAF-mutated melanoma cells (shades of red) and NRAS-mutated melanoma cell lines (shades of blue).

Figure S4

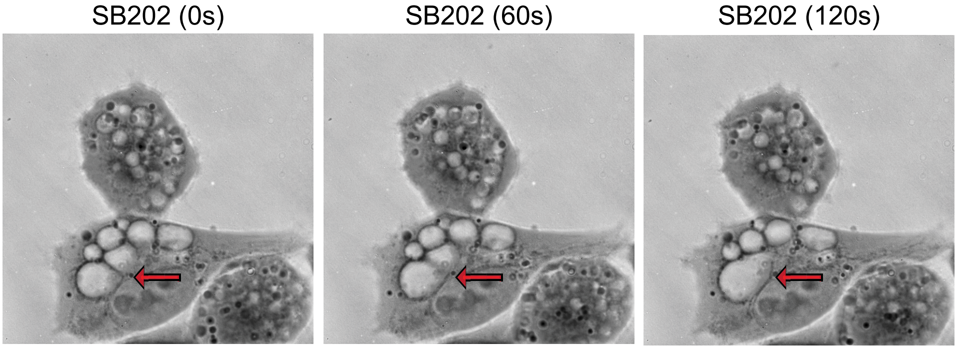

Supplementary figure S4. Coherence-controlled holographic microscopy. Coalescence of large vacuole-like vesicles was observed during live-cell imaging of A375 cells treated with SB202190 for 24 h. Three individual frames from a time-lapse recording (images acquired every 60s). The red arrow indicates the site of fusion.

Figure S5

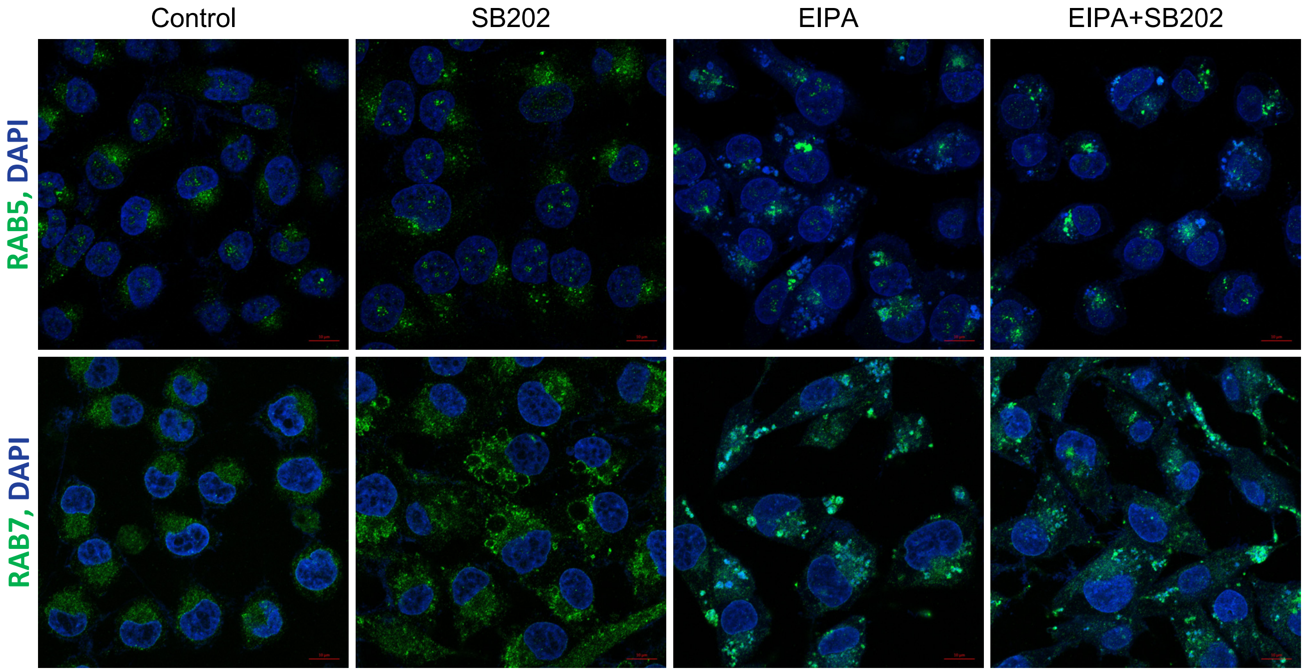

Supplementary figure S5. Immunofluorescence microscopy. Subcellular localization of endogenous RAB5 and RAB7 with DAPI in A375 melanoma cells treated for 20 hours with SB202190 (15µM) and EIPA (50µM), alone and in combination. Scale bar: 10µm.

Figure S6

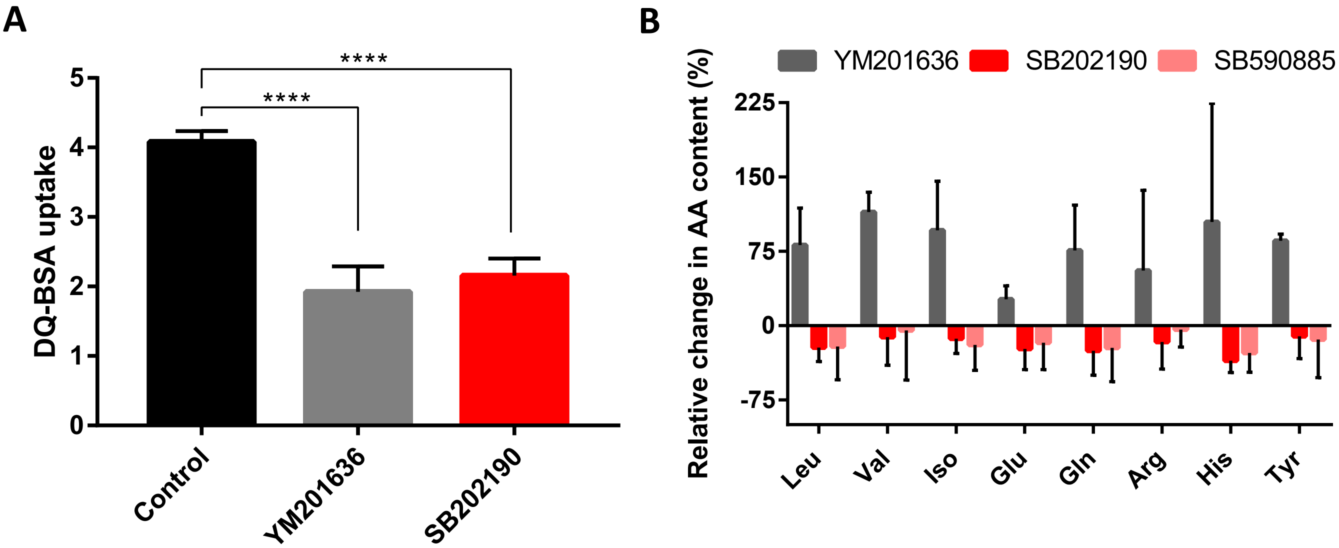

Supplementary figure S6. Nutrient uptake was negatively affected in A375 melanoma cells treated with pyridinyl imidazole compounds. (a) A375 cells were treated for one hour with DMSO (control), pyridinyl imidazole SB202190 (15μM), and PIKfyve inhibitor YM201636 (1μM), and DQ-BSA was added for 30 minutes. Then cells were chased in DQ-BSA free medium for 75 minutes, and the red fluorescence signal was analyzed by flow cytometry. Data from three independent analyses of DQ-BSA uptake are presented. **(b)** The NMR analysis of the content selected amino acids was performed in extracts of A375 melanoma cells treated for 14h with DMSO as a control, YM201636 (1μM), SB202190 (15μM), and SB590885 (5μM). Data were collected in three independent experiments.
