# Supplementary figures and images for: Dual Targeting of BRAF and mTOR Signaling in Melanoma Cells with Pyridinyl Imidazole Compounds
Source: Cancers (Basel). 2020 Jun 10;12(6):1516. doi: 10.3390/cancers12061516 (PMC7352453; doi:10.3390/cancers12061516)

Figure 1C

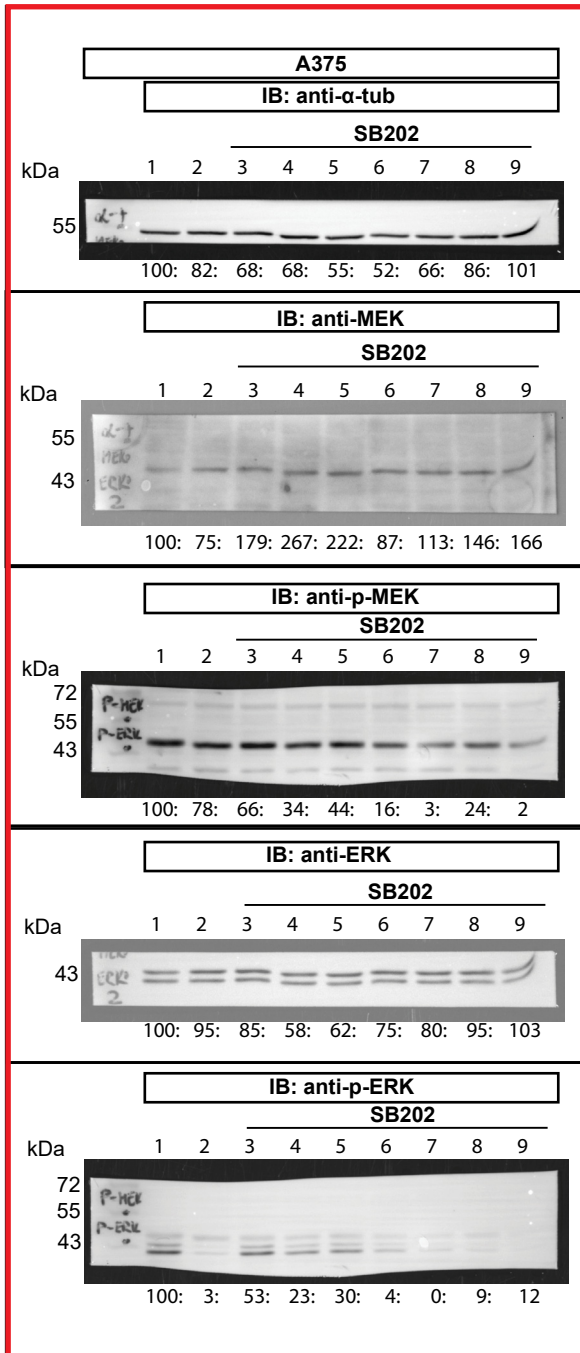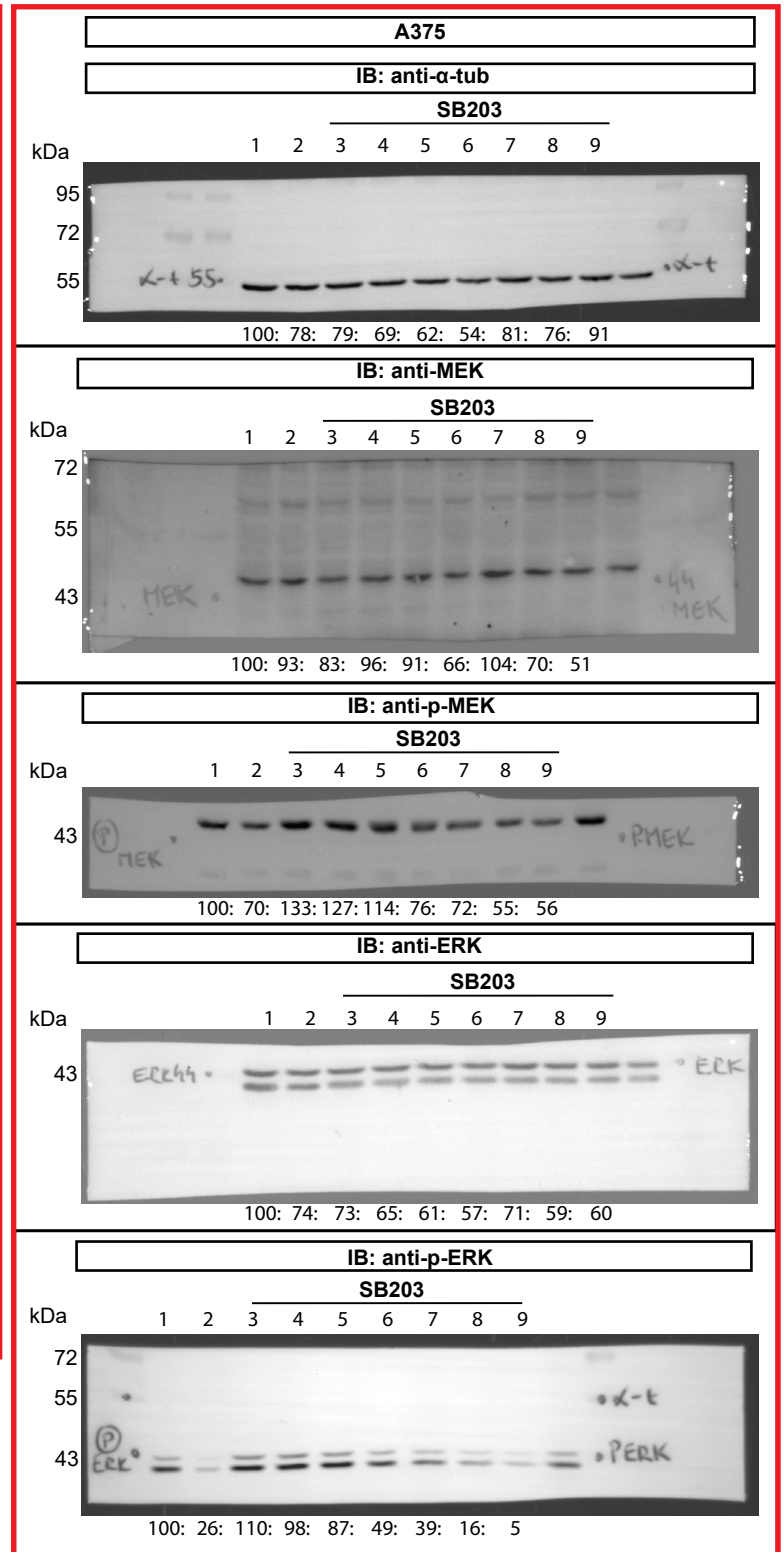

Figure 1D

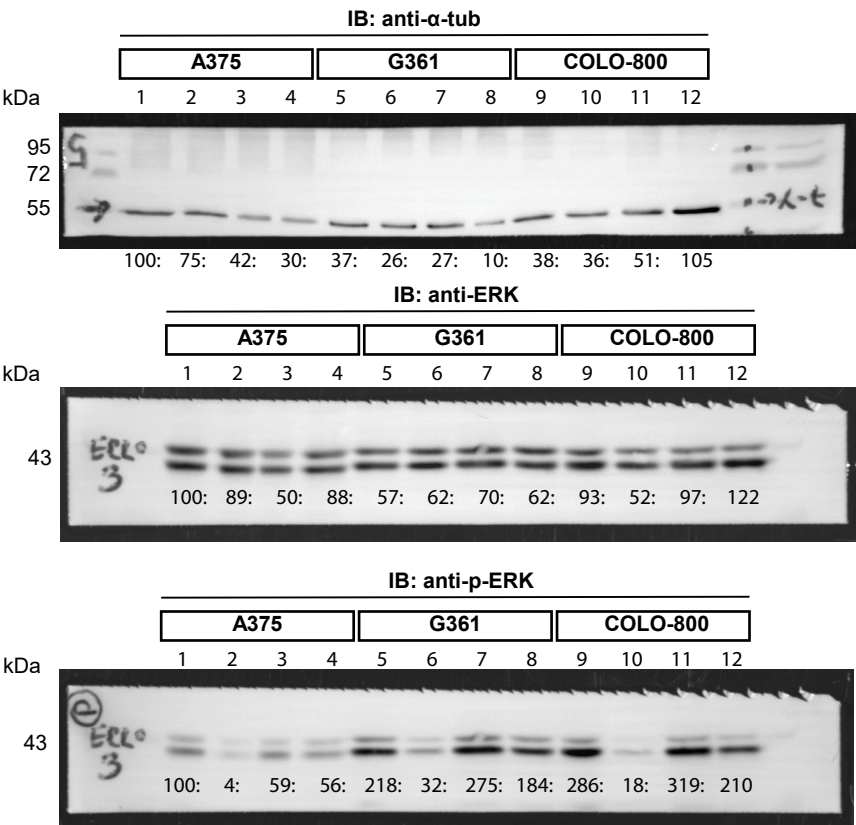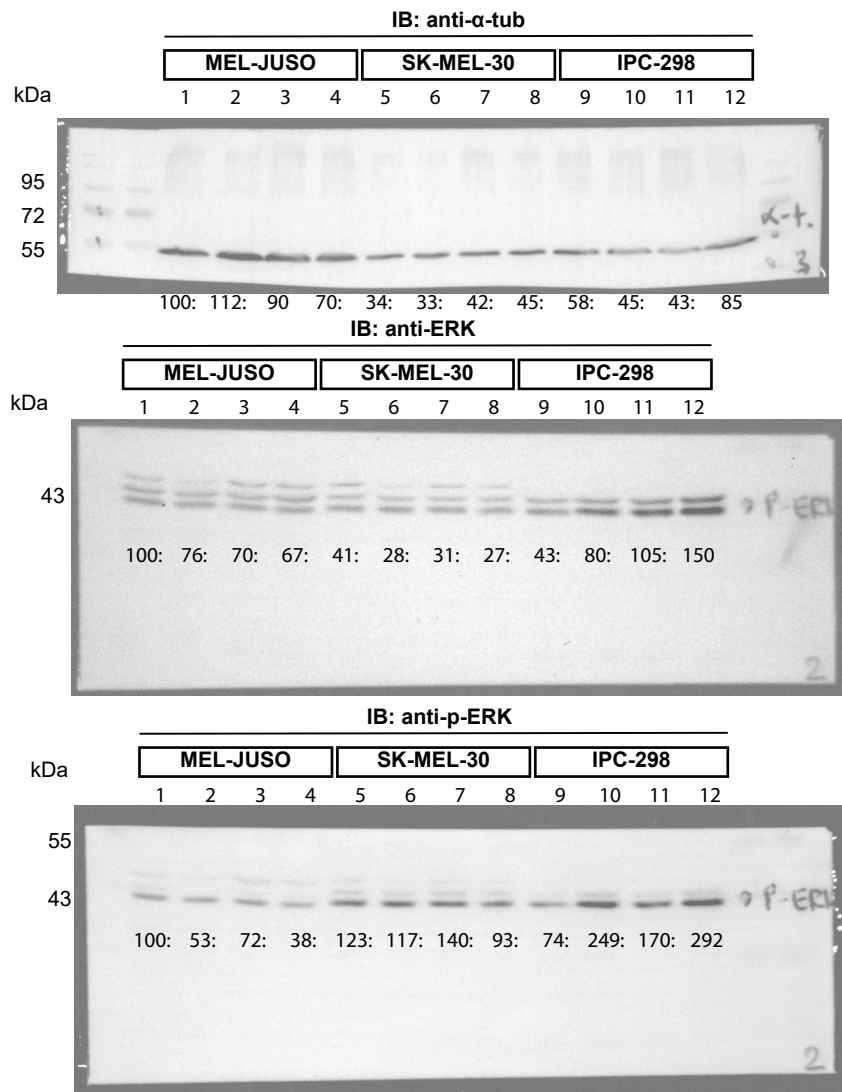

Figure 1E

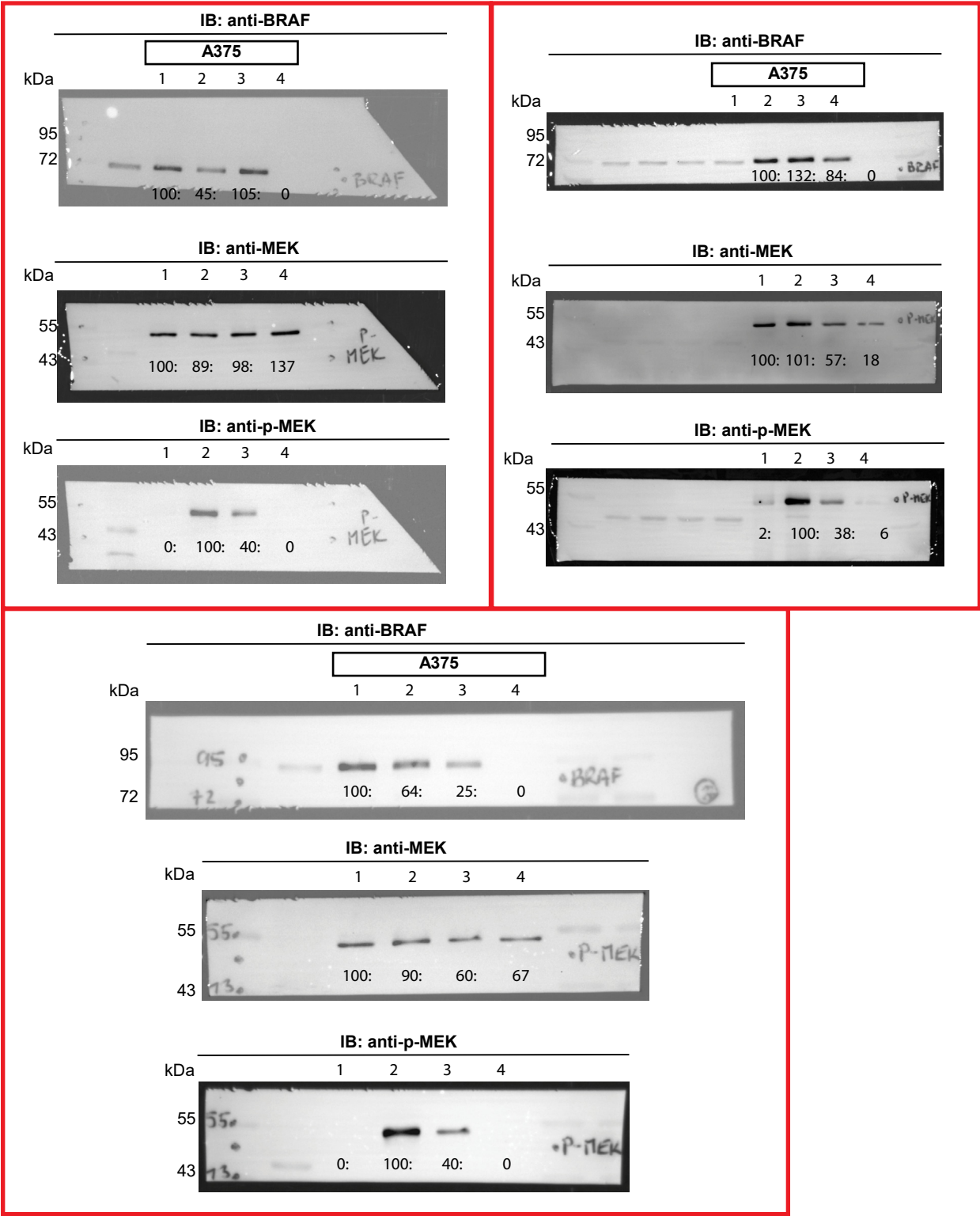

**Figure 6C**

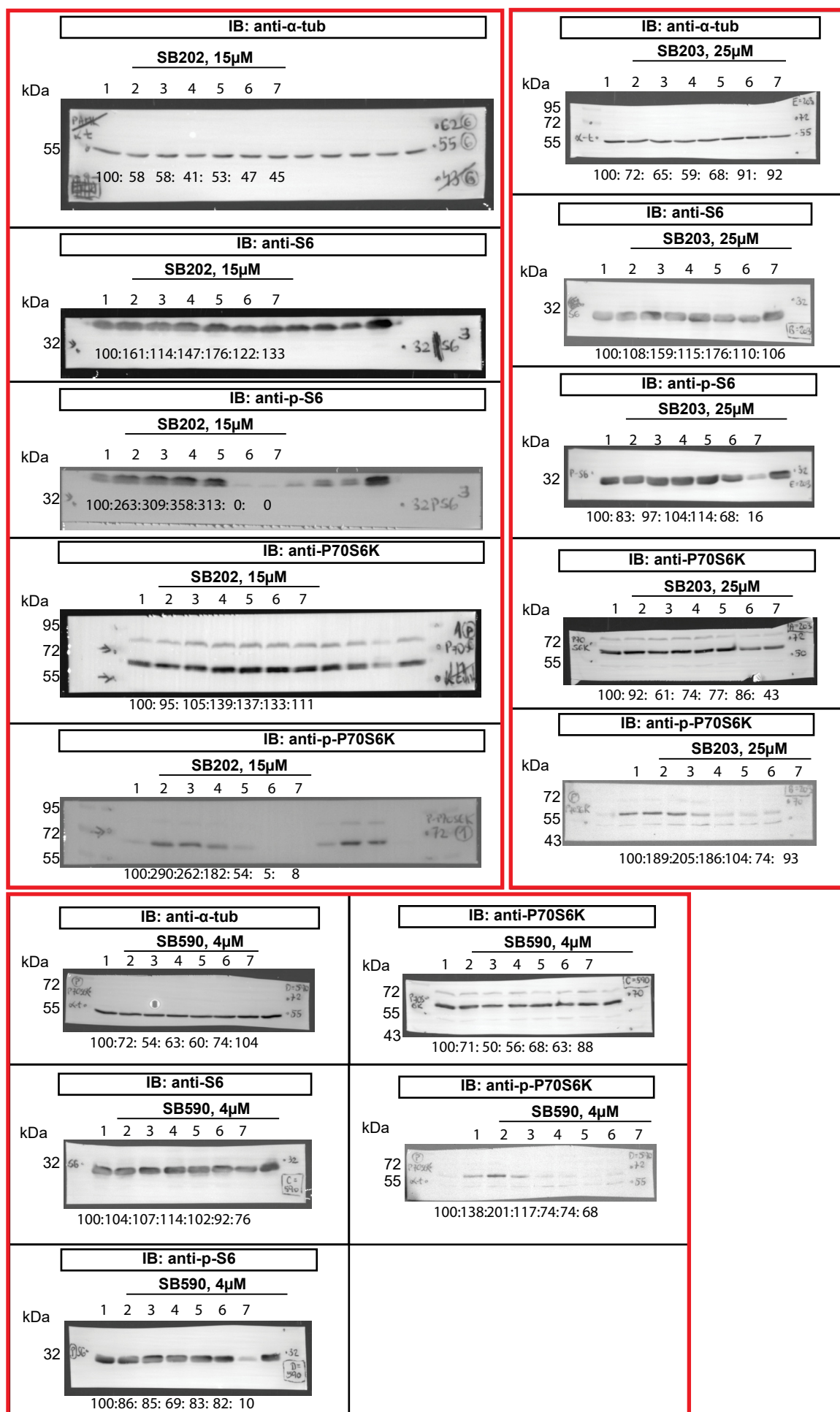

Figure 6E

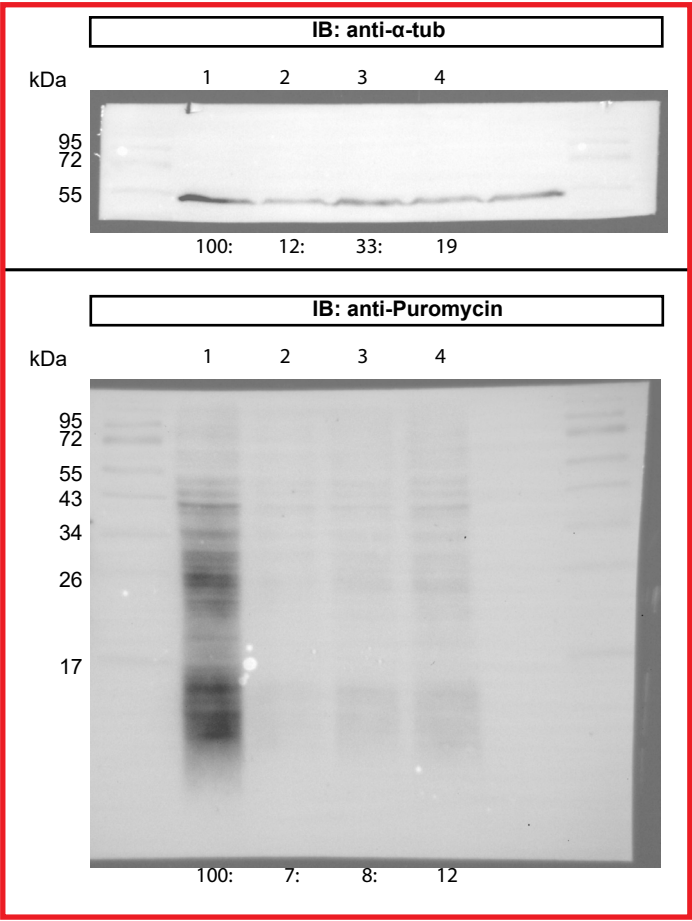

Supplement: Supplementary file 1 [file cancers-12-01516-s001.zip › Supplementary figure S7- uncropped WB.pdf]
